# Supplementary material for: Game theoretical inference of human behavior in social networks
Source: Nat Commun. 2019 Dec 3;10:5507. doi: 10.1038/s41467-019-13148-8 (PMC6890725; doi:10.1038/s41467-019-13148-8)
Supplement: Supplementary file 1 — Supplementary Information [file 41467_2019_13148_MOESM1_ESM.pdf]

Supplementary Information for "Game Theoretical Inference of Human Behaviour in Social Networks"

Nicolò Pagan et al.

Automatic Control Laboratory, ETH Zürich, Physikstrasse 3 8092 Zürich, Switzerland.

pagann@control.ee.ethz.ch

Supplementary Information for “Game Theoretical Inference of Human Behaviour in Social Networks”

Nicolò Pagan et al.

Automatic Control Laboratory, ETH Zürich, Physikstrasse 3 8092 Zürich, Switzerland.

pagann@control.ee.ethz.ch

Supplementary Note 1. *Theoretical Tools for Network Motifs*

Our stability notions involve two concepts: Nash equilibrium and Pareto optimality. The former is related with the selfish attitude of the individuals, the latter with their tendency to cooperation. Here, we discuss the tools we use to prove results on the necessary and sufficient conditions for equilibria of particular network architectures. First, we present an equivalent definition of Nash equilibrium and we draw a relation with the class of Variational Inequality problems. Second, we discuss the pairwise-Nash equilibrium concept concerning both of its aspects: the Nash condition and the Pareto optimality one.

Nash Equilibrium

A given network satisfies the Nash Equilibrium condition (C1) if the action of every agent belongs to her set of best responses. Formally, we define the best response set of an agent i as follows.

Definition 1 (Best Response)**.** *The set of best responses for player i, denoted as $BR_i(\mathbf{a}_{-i})$, is defined as*

$$BR_i(\mathbf{a}_{-i}) = \left\{ \arg\max_{\mathbf{a}_i \in \mathscr{A}} V_i(\mathbf{a}_i, \mathbf{a}_{-i}) \right\}.$$

This yields to the following equivalent definition of Nash equilibrium.

Definition 2. *The network $\mathscr{G}^\star$ is a Nash equilibrium if and only if for all agents i in $\mathscr{N}$,*

$$\mathbf{a}_i^\star \in BR_i(\mathbf{a}_{-i}^\star) = \left\{ \arg\max_{\mathbf{a}_i \in \mathscr{A}} V_i(\mathbf{a}_i, \mathbf{a}_{-i}^\star) \right\}. \qquad [1]$$

Thanks to this equivalent definition, we can use the Variational Inequality (VI) approach (for a complete survey, see[1]) in order to derive the necessary conditions certifying the Nash equilibrium property. We start by recovering the definition of VI problem.

Definition 3 (Variational Inequality Problem)**.** *The finite-dimensional Variational Inequality problem, $VI(F, \mathscr{X})$, is the problem of finding a vector $\mathbf{x}^\star \in \mathscr{X} \subset \mathbb{R}^n$, such that*

$$\langle F(\mathbf{x}^\star)^T, \mathbf{x} - \mathbf{x}^\star \rangle \le 0, \quad \forall \mathbf{x} \in \mathscr{X},$$

where F is a given continuous function from $\mathscr{X}$ to $\mathbb{R}^n$, $\mathscr{X}$ is a given closed, convex set, and $\langle \cdot\, , \cdot \rangle$ denotes the inner product in n-dimensional Euclidean space.

According to the VI theory[1], the following theorem holds.

Theorem 1. *If $\mathbf{x}^\star$ is a solution of*

$$\arg\max_{\mathbf{x} \in \mathscr{X}} f(\mathbf{x}), \qquad [2]$$

where f is continuously differentiable and $\mathscr{X}$ is closed and convex, then $\mathbf{x}^\star$ is a solution of

$$\langle \nabla f(\mathbf{x})\big|_{\mathbf{x}^\star}^T, \mathbf{x} - \mathbf{x}^\star \rangle \le 0, \quad \forall \mathbf{x} \in \mathscr{X}. \qquad [3]$$

Furthermore, if f is concave, then the solution sets of [2] and [3] coincide.

Coming back to our problem of characterizing Nash equilibria, we consider the payoff function $V_i(\mathbf{a}_i, \mathbf{a}_{-i})$, which is continuously differentiable. The action space $\mathscr{A} = [0,1]^{N-1}$ is closed and convex, then we can apply Theorem 1 in order to arrive at the following necessary condition.

Theorem 2 (NE necessary condition)**.** *If $\mathscr{G}^\star$ is a Nash equilibrium then for all agents i,*

$$\langle \nabla_{\mathbf{a}_i} V_i(\mathbf{a}_i, \mathbf{a}_{-i}^\star)\big|_{\mathbf{a}_i^\star}^T, \mathbf{a}_i - \mathbf{a}_i^\star \rangle \le 0, \quad \forall \mathbf{a}_i \in \mathscr{A}. \qquad [4]$$

Notice that each of the conditions [4] (one per agent) can be equivalently reduced to a set of $N-1$ inequalities in each agent's $(N-1)$-dimensional action space

$$
\begin{cases}
\left.\dfrac{\partial V_i\left(\mathbf{a}_i,\mathbf{a}^\star_{-i}\right)}{\partial a_{i1}}\right|_\star (a_{i1}-a^\star_{i1}) \le 0, & \forall a_{i1}\in[0,1]\\
\quad\vdots & \\
\left.\dfrac{\partial V_i\left(\mathbf{a}_i,\mathbf{a}^\star_{-i}\right)}{\partial a_{i,i-1}}\right|_\star \left(a_{i,i-1}-a^\star_{i,i-1}\right) \le 0, & \forall a_{i,i-1}\in[0,1]\\
\left.\dfrac{\partial V_i\left(\mathbf{a}_i,\mathbf{a}^\star_{-i}\right)}{\partial a_{i,i+1}}\right|_\star \left(a_{i,i+1}-a^\star_{i,i+1}\right) \le 0, & \forall a_{i,i+1}\in[0,1]\\
\quad\vdots & \\
\left.\dfrac{\partial V_i\left(\mathbf{a}_i,\mathbf{a}^\star_{-i}\right)}{\partial a_{iN}}\right|_\star (a_{iN}-a^\star_{iN}) \le 0, & \forall a_{iN}\in[0,1],
\end{cases}
\tag{5}
$$

where we used the following shorthand notation $f|_\star = f\left(\mathbf{a}^\star_i\right)$. Certainly, [5] implies [4], as the sum of non-positive quantities is non-positive. Conversely, [4] implying [5] is obtained by consecutively taking $\mathbf{a}_i$ such that all the components but the j-th are equal to $a^\star_{ij}$. Thus, all but the j-th contribution in [4] become zero, leading to the j-th equation in [5].

An explicit parametric necessary condition for NE can then be derived by using the expression of the payoff function, as in the next theorem.

Theorem 3 (NE explicit necessary condition)**.** *If $\mathscr{G}^\star$ is a Nash equilibrium then for each agent i and for each $j \neq i$, the equilibrium strategy $a^\star_{ij}$ must be such that, for any other strategy $a_{ij}\in[0,1]$*

$$
\begin{aligned}
&\Bigg[\alpha\delta\left(a^\star_{ji}\left(1+\delta\sum_{k\neq i}a^\star_{ki}\right)+\delta\sum_{k\neq j,i}a^\star_{jk}a^\star_{ki}\right)+\\
&+\beta\left(\sum_{k\neq i,j}a^\star_{ik}\left(a^\star_{kj}+a^\star_{jk}\right)\right)-\gamma\Bigg]\cdot\left(a_{ij}-a^\star_{ij}\right)\le 0.
\end{aligned}
\tag{6}
$$

Proof. We start by recalling the definition of the payoff function V_i of a generic agent i.

$$
\begin{aligned}
&V_i(\mathbf{a}_i,\mathbf{a}_{-i}) =\\
&\alpha\cdot\underbrace{\left(\sum_k a_{ki}+\delta\sum_l\sum_k a_{lk}a_{ki}+\delta^2\sum_m\sum_l\sum_k a_{ml}a_{lk}a_{ki}\right)}_{t_i(\mathbf{a}_i,\mathbf{a}_{-i})}+\\
&+\beta\cdot\underbrace{\left(\sum_k a_{ik}\left(\sum_l a_{il}a_{lk}\right)\right)}_{u_i(\mathbf{a}_i,\mathbf{a}_{-i})}-\gamma\cdot\underbrace{\left(\sum_k a_{ik}\right)}_{c_i(\mathbf{a}_i)}.
\end{aligned}
$$

We firstly consider the centrality term $t_i(\mathbf{a}_i,\mathbf{a}_{-i})$, and we rewrite it in a form which allows identifying $a_{i\bullet}$'s contribution

$$
\begin{aligned}
t_i(\mathbf{a}_i,\mathbf{a}_{-i}) &= \sum_k a_{ki}+\delta\sum_l\sum_k a_{lk}a_{ki}+\\
&+\delta^2\sum_m\sum_l\sum_k a_{ml}a_{lk}a_{ki} =\\
&=\sum_{k\neq i}a_{ki}+\underbrace{\cancel{a_{ii}}}_{=0}+\delta\left(\sum_{l\neq i}\sum_{k\neq i}a_{lk}a_{ki}\right)\\
&+\delta\left(\sum_{k\neq i}a_{ik}a_{ki}+\cancel{\sum_{l\neq i}a_{li}\underbrace{a_{ii}}_{=0}}+\underbrace{\cancel{a^2_{ii}}}_{=0}\right)+\\
&+\delta^2\left(\sum_{m\neq i}\sum_{l\neq i}\sum_{k\neq i}a_{ml}a_{lk}a_{ki}+\sum_{l\neq i}\sum_{k\neq i}a_{il}a_{lk}a_{ki}\right)+\\
&+\delta^2\left(\sum_{m\neq i}\sum_{k\neq i}a_{mi}a_{ik}a_{ki}+\cancel{\sum_{m\neq i}\sum_{l\neq i}a_{ml}a_{lk}\underbrace{a_{ii}}_{=0}}\right)+\\
&+\delta^2\left(\cancel{\sum_{m\neq i}a_{mi}\underbrace{a^2_{ii}}_{=0}}\right)+\\
&+\delta^2\left(\cancel{\sum_{l\neq i}a_{il}a_{li}\underbrace{a_{ii}}_{=0}}+\cancel{\sum_{k\neq i}\underbrace{a_{ii}}_{=0}a_{ik}a_{ki}}+\underbrace{\cancel{a^3_{ii}}}_{=0}\right).
\end{aligned}
$$

By simplifying and rearranging the terms, it is possible to isolate the $a_{i\bullet}$'s contribution in the summation, obtaining the following expression for the centrality term $t_i(\mathbf{a}_i,\mathbf{a}_{-i})$

$$
\begin{aligned}
&\underbrace{\sum_{k\neq i}a_{ki}+\delta\left(\sum_{l\neq i}\sum_{k\neq i}a_{lk}a_{ki}\right)+\delta^2\left(\sum_{m\neq i}\sum_{l\neq i}\sum_{k\neq i}a_{ml}a_{lk}a_{ki}\right)}_{f(\mathbf{a}_{-i},\delta)}\\
&+\delta\sum_{k\neq i}a_{ik}a_{ki}+\delta^2\left(\sum_{m\neq i}\sum_{k\neq i}a_{mi}a_{ik}a_{ki}+\sum_{l\neq i}\sum_{k\neq i,l}a_{il}a_{lk}a_{ki}\right)\\
&+\delta^2\left(\cancel{\sum_{l\neq i}a_{il}\underbrace{a_{ll}}_{=0}a_{li}}\right)=\\
&=f(\mathbf{a}_{-i},\delta)+\delta\left(\sum_{k\neq i}a_{ik}a_{ki}\right)\left(1+\delta\sum_{m\neq i}a_{mi}\right)+\\
&+\delta^2\left(\sum_{l\neq i}a_{il}\sum_{k\neq i,l}a_{lk}a_{ki}\right),
\end{aligned}
$$

where $f(\mathbf{a}_{-i},\delta)$ denotes the contribution which only depends on $\mathbf{a}_{-i}$ and not on the decision variables $a_{i\bullet}$.

We then consider the other two contributions, namely the clustering coefficient $u_i(\mathbf{a}_i,\mathbf{a}_{-i})$ and the cost $c_i(\mathbf{a}_i)$. By using the non-reflexive property, i.e., $a_{ii}=0$ for all i, we obtain

$$
u_i(\mathbf{a}_i,\mathbf{a}_{-i})=\sum_{k\neq i}a_{ik}\left(\sum_{l\neq i,k}a_{il}a_{lk}\right),\qquad c_i(\mathbf{a}_i)=\left(\sum_{k\neq i}a_{ik}\right).
$$

We can now compute the partial derivative of the payoff function $V_i(a_i,\mathbf{a}_{-i})$ with respect to the decision variables a_{ij}, for all $j\neq i$,

and evaluate it at the equilibrium strategy

$$\left.\frac{\partial V_i(\mathbf{a}_i,\mathbf{a}^\star_{-i})}{\partial a_{ij}}\right|_\star = \alpha\delta\left(a^\star_{ji}\left(1+\delta\sum_{k\neq i}a^\star_{ki}\right)+\delta\sum_{k\neq j,i}a^\star_{jk}a^\star_{ki}\right)+ \\ +\beta\left(\sum_{k\neq i,j}a^\star_{ik}\left(a^\star_{kj}+a^\star_{jk}\right)\right)-\gamma.$$

Finally, we can conclude that the necessary condition [4] is equivalent to the following: for each agent i, for each $j\neq i$, for each $a_{ij}\in[0,1]$

$$\left[\alpha\delta\left(a^\star_{ji}\left(1+\delta\sum_{k\neq i}a^\star_{ki}\right)+\delta\sum_{k\neq j,i}a^\star_{jk}a^\star_{ki}\right)+ \right. \\ \left. +\beta\left(\sum_{k\neq i,j}a^\star_{ik}\left(a^\star_{kj}+a^\star_{jk}\right)\right)-\gamma\right]\cdot\left(a_{ij}-a^\star_{ij}\right)\leq 0.$$

The latter equals condition [6]. □

Remark 1. Unfortunately, it is not possible to draw conclusions on the sufficiency of the condition. From Theorem 1, this would require the payoff function to be concave but unfortunately that is not necessarily the case, due to the clustering coefficient. Indeed, one can compute the Hessian matrix H_i of the payoff function of agent i, evaluated at $\mathbf{a}^\star_i$, obtaining, for each component (j,k), with $j,k\neq i$,

$$(H^\star_i)_{jk}=\left.\frac{\partial^2 V_i(\mathbf{a}_i,\mathbf{a}^\star_{-i})}{\partial a_{ij}\partial a_{ik}}\right|_\star=\begin{cases}\beta\left(a^\star_{jk}+a^\star_{kj}\right), \forall j\neq k\\ 0, \forall j=k.\end{cases}$$

Depending on the topology, $H^\star_i$ can satisfy different properties. For instance, in the case of the empty network, $\mathbf{a}^\star_{-i}=\mathbf{0}_{(N-1)\times(N-1)}, \forall i$, therefore for all agents i, $H^\star_i=\mathbf{0}_{(N-1)\times(N-1)}$ is negative semi-definite, and V_i is concave. In this case, necessary and sufficient conditions coincide, as we will see in Theorem 7. Conversely, in the case of the complete network $\mathbf{a}^\star_{-i}=\mathbf{1}_{(N-1)\times(N-1)}, \forall i$, $H^\star_i$ reads as

$$H^\star_i=\begin{bmatrix}0 & 2\beta & 2\beta & \cdots & 2\beta\\ 2\beta & 0 & 2\beta & \cdots & 2\beta\\ \vdots & & 0 & & \vdots\\ \vdots & & & \ddots & 2\beta\\ 2\beta & \cdots & & 2\beta & 0\end{bmatrix}.$$

The spectrum of $H^\star_i$ is $spec(H^\star_i)=\{-2\beta, 2\beta*(N-2)\}$, implying that $H^\star_i$ is indefinite (unless $\beta=0$). As Theorem 8 will show, in this case the necessary condition is not sufficient.

Pairwise-Nash equilibrium

Similarly to what just done for the Nash equilibrium condition, the VI approach can be used to derive some necessary conditions related to the Nash property (C2) of the pairwise-Nash stability notion. In order to do so, we first note that (C2) is equivalent to the following formulation: for all agents i, for all agents j different from i, $a^\star_{ij}$ is such that

$$a^\star_{ij}\in\left\{\arg\max_{a_{ij}\in[0,1]} V_i(a_{ij},\mathbf{a}^\star_{i-(i,j)},\mathbf{a}^\star_{-i})\right\}. \quad [7]$$

By applying the VI theory we obtain a similar result on the necessary condition for (C2). Note that [7] differs from [1] in the action space. In particular, one can show that the Nash equilibrium condition [1] implies [7]. Indeed, the Nash equilibrium equivalent definition requires that, for each agent i, the equilibrium strategy $\mathbf{a}^\star_i=\left[a^\star_{i1},\ldots,a^\star_{i,i-1},a^\star_{i,i+1},\ldots,a_{iN}\right]$ solves the optimization problem [1]. Therefore, if $\mathbf{a}^\star_i$ satisfies [1], certainly each of its components $a^\star_{ij}$ satisfies the same optimization problem when all the other components a_{ik}, with k different from j, are equal to $a^\star_{ik}$ (in other words, when $\mathbf{a}_{i-(i,j)}=\mathbf{a}^\star_{i-(i,j)}$). Thus, if $\mathbf{a}^\star_i$ solves the optimization problem [1], it also satisfies [7]. Thanks to this observation, we have that (C1) implies (C2).

Theorem 4 (PNE, necessary condition for (C2))**.** *If $\mathscr{G}^\star$ is a pairwise-Nash equilibrium, then for each agent i and for each $j\neq i$, the equilibrium strategy $a^\star_{ij}$ must be such that, for any other strategy $a_{ij}\in[0,1]$*

$$\left.\frac{\partial V_i\left(a_{ij},\mathbf{a}^\star_{i-(i,j)},\mathbf{a}^\star_{-i}\right)}{\partial a_{ij}}\right|_\star\left(a_{ij}-a^\star_{ij}\right)\leq 0. \quad [8]$$

Proof. Consider the equivalent definition of the Nash property (C2) as in [7]. Applying Theorem 1 for each agent i and for each agent j different from i yields to the following necessary condition: $a^\star_{ij}$ must satisfy

$$\left.\frac{\partial V_i\left(a_{ij},\mathbf{a}^\star_{i-(i,j)},\mathbf{a}^\star_{-i}\right)}{\partial a_{ij}}\right|_\star\left(a_{ij}-a^\star_{ij}\right)\leq 0, \forall a_{ij}\in[0,1].$$

□

Moreover, such a VI result turns out to be necessary and sufficient for optimality in [7], due to the fact that the action space of each agent i is restricted to one link at a time, and thus the objective function in [7] is linear in the decision variable, and therefore concave. This is shown in the next theorem.

Theorem 5 (PNE, necessary and sufficient condition for (C2))**.** *If $\mathscr{G}^\star$ satisfies condition (C2) then, for each agent i and for each $j\neq i$, $a^\star_{ij}$ must be such that, for each $a_{ij}\in[0,1]$*

$$\left[\alpha\delta\left(a^\star_{ji}\left(1+\delta\sum_{k\neq i}a^\star_{ki}\right)+\delta\sum_{k\neq j,i}a^\star_{jk}a^\star_{ki}\right)+ \right. \\ \left. +\beta\left(\sum_{k\neq i,j}a^\star_{ik}\left(a^\star_{kj}+a^\star_{jk}\right)\right)-\gamma\right]\cdot\left(a_{ij}-a^\star_{ij}\right)\leq 0. \quad [9]$$

Conversely, if [9] holds, then (C2) is satisfied.

Proof. In order to prove the result, we first recover an explicit formulation for [8] from Thorem 3 and show that the payoff function is concave. Then, we apply the previous theorem using the VI theory result in Theorem 1 on the sufficiency of the conditions.

Isolating the contribution of the variable a_{ij} from those of $\mathbf{a}_{i-(i,j)}$ and $\mathbf{a}_{-i}$ in the payoff function of agent i we get

$$
\begin{aligned}
&V_i(a_{ij},\mathbf{a}_{i-(i,j)},\mathbf{a}_{-i}) = \\
&= \alpha\left[f(\mathbf{a}_{-i}) + \delta\left(\sum_{k\neq i,j} a_{ik}a_{ki} + a_{ij}a_{ji}\right)\left(1+\delta\sum_{m\neq i} a_{mi}\right) + \right.\\
&\left. +\delta^2\left(\sum_{l\neq i,j} a_{il}\sum_{k\neq i,l}(a_{lk}a_{ki}) + a_{ij}\sum_{k\neq i,j}(a_{jk}a_{ki})\right)\right] + \\
&+\beta\left[\sum_{k\neq i,j} a_{ik}\left(\sum_{l\neq i,k,j} a_{il}a_{lk}\right) + \sum_{k\neq i,j} a_{ik}a_{ij}a_{jk} + a_{ij}\sum_{l\neq i,j} a_{il}a_{lj}\right] + \\
&-\gamma\left[\sum_{k\neq i,j} a_{ik} + a_{ij}\right].
\end{aligned}
$$

Then, after some easy algebraic manipulation, we have

$$
\begin{aligned}
&V_i(a_{ij},\mathbf{a}_{i-(i,j)},\mathbf{a}_{-i}) = \\
&= \alpha\cdot f(\mathbf{a}_{-i}) + \underbrace{\alpha\delta\left(\sum_{k\neq i,j} a_{ik}a_{ki}\right)\left(1+\delta\sum_{m\neq i} a_{mi}\right)}_{(*)} + \\
&\underbrace{+\alpha\delta^2\left(\sum_{l\neq i,j} a_{il}\sum_{k\neq i,l}(a_{lk}a_{ki})\right) + \beta\left(\sum_{k\neq i,j} a_{ik}\left(\sum_{l\neq i,k,j} a_{il}a_{lk}\right)\right)}_{(**)} + \\
&\underbrace{-\gamma\left(\sum_{k\neq i,j} a_{ik}\right)}_{(***)} + \alpha\delta a_{ij}a_{ji}\left(1+\delta\sum_{m\neq i} a_{mi}\right) + \alpha\delta^2 a_{ij}\sum_{k\neq i,j}(a_{jk}a_{ki}) + \\
&+\beta a_{ij}\sum_{k\neq i,j} a_{ik}\left(a_{jk}+a_{kj}\right) - \gamma a_{ij}.
\end{aligned}
$$

Notice that $(*), (**), (***)$ are not functions of the action variable a_{ij}. Thus, the gradient of the payoff function reads as

$$
\begin{aligned}
\frac{\partial V_i(a_{ij},\mathbf{a}_{i-(i,j)},\mathbf{a}_{-i})}{\partial a_{ij}} &= \alpha\delta a_{ji}\left(1+\delta\sum_{m\neq i} a_{mi}\right) + \\
&+\alpha\delta^2\sum_{k\neq i,j}(a_{jk}a_{ki}) + \\
&+\beta\sum_{k\neq i,j} a_{ik}\left(a_{jk}+a_{kj}\right) - \gamma.
\end{aligned}
$$

Note that the gradient is constant with respect to the variable a_{ij}. Consequently, the objective function $V_i(a_{ij},\mathbf{a}^\star_{i-(i,j)},\mathbf{a}^\star_{-i})$ is linear in a_{ij}, thus concave. Applying the previous theorem leads precisely to the necessary condition [9]. Moreover, as the objective function is concave, [9] is a necessary and sufficient condition for the Nash property (C2) of the pairwise-Nash definition.

□

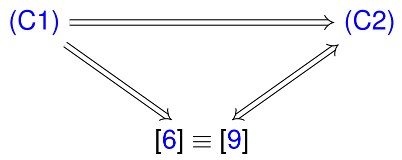

Supplementary Figure 1. Scheme of dependencies of Nash equilibrium conditions.

Remark 2. It is worth to highlight that the necessary conditions [6] and [9] derived respectively in Theorems 3 and 5, for the two different notions of equilibria, coincide. However, due to the different action space definition, [9] is a sufficient condition for (C2), while [6] is not a sufficient condition for (C1). The scheme of dependencies is shown in Supplementary Fig. 1.

Before proving the main results, we finally prove a useful theorem on a sufficient condition for the Pareto optimality property of the pairwise-Nash equilibrium, namely condition (C3). Let us first review the definition of Pareto optimality in economics (see[2]).

Definition 4. *Consider an economy with n agents and k goods. Then an allocation $x = \{x_1,\dots,x_n\}$, where $x_i \in \mathbb{R}^k$ for all i, is Pareto optimal if there is no other feasible allocation $\{x'_1,\dots,x'_n\}$ such that, for utility function u_i for each agent i, $u_i(x'_i) \geq u_i(x_i)$ for all $i \in \{1,\dots,n\}$ with $u_i(x'_i) > u_i(x_i)$ for some i.*
Moreover, the set of all Pareto optimal allocations constitutes the Pareto optimal front.

In other words, the Pareto optimality condition requires that there exists no allocation of goods which strictly increases the payoff of at least one agent while not decreasing the payoff of the others. On the other hand, condition (C3) of the pairwise-Nash equilibrium requires that for all pairs (i,j), and for all pairs (a_{ij},a_{ji}) in $[0,1]^2$,

$$
\begin{gathered}
V_i\left(a_{ij},a_{ji},\mathbf{a}^\star_{-(i,j)}\right) > V_i\left(a^\star_{ij},a^\star_{ji},\mathbf{a}^\star_{-(i,j)}\right)\\
\Downarrow\\
V_j\left(a_{ij},a_{ji},\mathbf{a}^\star_{-(i,j)}\right) < V_j\left(a^\star_{ij},a^\star_{ji},\mathbf{a}^\star_{-(i,j)}\right).
\end{gathered}
$$

In other words, it is satisfied if there exists no other pair (a_{ij},a_{ji}) in $[0,1]^2$ such that i and j are simultaneously better off, with at least one of the two being strictly better off. Thus, Condition (C3) in fact corresponds to a Pareto Optimality condition.

Theorem 6 (Pareto Optimality sufficient condition)**.** *Let $\mathscr{G}^\star$ be a network.*

(i) If (C2) is satisfied for a pair of agents (i,j) and $a^\star_{ij} = a^\star_{ji} = 1$, then (C3) is satisfied for the same pair;

(ii) if for all pairs (i,j), the pair $(a^\star_{ij},a^\star_{ji})$ satisfies for all pairs $(a_{ij},a_{ji}) \in [0,1]^2$,

$$
\begin{aligned}
&V_i\left(a_{ij},a_{ji},\mathbf{a}^\star_{-(i,j)}\right) + V_j\left(a_{ij},a_{ji},\mathbf{a}^\star_{-(i,j)}\right) \leq \\
&V_i\left(a^\star_{ij},a^\star_{ji},\mathbf{a}^\star_{-(i,j)}\right) + V_j\left(a^\star_{ij},a^\star_{ji},\mathbf{a}^\star_{-(i,j)}\right),
\end{aligned}
$$

then the Pareto Optimality condition (C3) is satisfied.

Proof. (i) The Nash equilibrium condition (C2) for agents i and j requires

$$V_i\left(a_{ij},\mathbf{a}^\star_{i-(i,j)},\mathbf{a}^\star_{-i}\right)\leq V_i\left(a_{ij}{}^\star,\mathbf{a}^\star_{i-(i,j)},\mathbf{a}^\star_{-i}\right),\ \forall a_{ij}\in[0,1]$$
$$V_j\left(a_{ji},a^\star_{j-(j,i)},\mathbf{a}^\star_{-j}\right)\leq V_j\left(a_{ji}{}^\star,a^\star_{j-(j,i)},\mathbf{a}^\star_{-j}\right),\ \forall a_{ji}\in[0,1].$$

We consider the payoff of agent i as a function of a meeting strategy (a_{ij},a_{ji}), namely $V_i\left(a_{ij},a_{ji},\mathbf{a}^\star_{-(i,j)}\right)$, and we take the derivative with respect to a_{ji}

$$\begin{aligned}\frac{\partial V_i\left(a_{ij},a_{ji},\mathbf{a}^\star_{-(i,j)}\right)}{\partial a_{ji}} &= \alpha\frac{\partial t_i\left(a_{ij},a_{ji},\mathbf{a}^\star_{-(i,j)}\right)}{\partial a_{ji}}+\\ &+\beta\frac{\partial u_i\left(a_{ij},a_{ji},\mathbf{a}^\star_{-(i,j)}\right)}{\partial a_{ji}}+\\ &-\gamma\frac{\partial c_i\left(a_{ij},a_{ji},\mathbf{a}^\star_{-(i,j)}\right)}{\partial a_{ji}}=\\ &+\alpha\frac{\partial t_i\left(a_{ij},a_{ji},\mathbf{a}^\star_{-(i,j)}\right)}{\partial a_{ji}}+\\ &+\cancel{\beta\frac{\partial}{\partial a_{ji}}\left(\sum_{k\neq i}a_{ik}\left(\sum_{l\neq i,k}a_{il}a^\star_{lk}\right)\right)}+\\ &-\cancel{\gamma\frac{\partial}{\partial a_{ji}}\left(\sum_{k\neq i}a_{ik}\right)}\geq 0,\end{aligned}$$

where we used the fact that the clustering coefficient u_i and the cost c_i do not depend on a_{ji}, and that t_i is a non-decreasing function with respect to all its arguments, being a sum of weighted paths. Then, we have for all a_{ij}

$$\begin{aligned}V_i\left(a_{ij},a_{ji},\mathbf{a}^\star_{-(i,j)}\right)&\leq V_i\left(a_{ij},1,\mathbf{a}^\star_{-(i,j)}\right)=\\ &=V_i\left(a_{ij},a^\star_{ji},\mathbf{a}^\star_{-(i,j)}\right)=\\ &=V_i\left(a_{ij},\mathbf{a}^\star_{i-(i,j)},\mathbf{a}^\star_{-i}\right).\end{aligned}\qquad[10]$$

We recall condition (C2): for each agent i, for each agent j and for each possible $a_{ij}\in[0,1]$,

$$V_i\left(a_{ij},\mathbf{a}^\star_{i-(i,j)},\mathbf{a}^\star_{-i}\right)\leq V_i\left(a^\star_{ij},\mathbf{a}^\star_{i-(i,j)},\mathbf{a}^\star_{-i}\right).$$

Combining [10] and (C2) yields

$$V_i\left(a_{ij},a_{ji},\mathbf{a}^\star_{-(i,j)}\right)\overset{[10]}{\leq}V_i\left(a_{ij},\mathbf{a}^\star_{i-(i,j)},\mathbf{a}^\star_{-i}\right)\overset{(C2)}{\leq}V_i\left(a_{ij}{}^\star,a^\star_{ji},\mathbf{a}^\star_{-(i,j)}\right).$$

The same reasoning holds for j. Therefore, neither i nor j can be strictly better off, thus condition (C3) is satisfied.

(ii) We prove this by contraposition. Assume that (C3) is not satisfied. Then necessarily there exists a meeting strategy (a_{ij},a_{ji}) such that

$$V_i\left(a_{ij},a_{ji},\mathbf{a}^\star_{-(i,j)}\right)>V_i\left(a_{ij}{}^\star,a^\star_{ji},\mathbf{a}^\star_{-(i,j)}\right),$$
$$V_j\left(a_{ij},a_{ji},\mathbf{a}^\star_{-(i,j)}\right)>V_j\left(a_{ij}{}^\star,a^\star_{ji},\mathbf{a}^\star_{-(i,j)}\right).$$

By summing up the two equations, we immediately obtain a contradiction with the statement of the theorem.

□

Supplementary Note 2. Results on Network Motifs

In this section, we present the proof of the core results of our study on network motifs, namely we focus on deriving necessary and sufficient parametric conditions which guarantee the equilibrium of different network architectures. The results cover very diverse networks: firstly, we consider the empty and the complete graph, which are networks in which either no link exists or every possible link between two distinct agents has full weight. Secondly, we analyze two examples of bipartite networks, i.e., networks where nodes can be partitioned into two sets such that only links across the two sets exist. Among all the possible graphs with this structure, we study the balanced complete bipartite network (where nodes are evenly split into two factions, and each node is connected with all the nodes in the opposite faction), and the most unbalanced network, i.e., the star network (where a central agent is connected with all the other nodes, and vice versa).

Empty Network

We then first recall the definition of the empty graph and subsequently we present the theorem on the parametric condition for its equilibrium.

Definition (Empty Network)**.** *We call empty network (EN) a graph $\mathscr{G}^{EN}$ of N nodes such that there exists no link with positive weight between any two agents. In other words, $a_{ij}=0$ for all pairs (i,j).*

Theorem 7. *Let $\mathscr{G}^{EN}$ be an empty network. Then*

a) $\mathscr{G}^{EN}$ is always a NE,

b) $\mathscr{G}^{EN}$ is a PNE if and only if $\gamma\geq\alpha\left(1+\delta+\delta^2\right)$.

Proof. a) We start by considering NE stability. Given the symmetry of the topology, it is sufficient to consider a generic agent i.

($\Longrightarrow$): We invoke Theorem 3, and we derive the necessary condition in the form of [6], applied to the empty network

$$0\cdot(a_{ij}-0)\leq 0,\quad\forall a_{ij}\in[0,1],$$

which reduces to a trivial identity. Therefore, there are no necessary conditions for Nash equilibrium stability.

($\Longleftarrow$): We prove sufficiency by verifying the Nash equilibrium definition, i.e. by checking that, for any action $\mathbf{a}_i\in\mathscr{A}$, it holds that

$$V_i(\mathbf{a}_i,\mathbf{a}^\star_{-i})\leq V_i(\mathbf{a}^\star_i,\mathbf{a}^\star_{-i}).$$

Note that $V_i^\star = V_i(\mathbf{a}_i^\star, \mathbf{a}_{-i}^\star) = 0$, as there are no links in the network. On the other hand, for any generic action $a_i \in \mathscr{A}$,

$$V_i(\mathbf{a}_i, \mathbf{a}_{-i}^\star) = 0 - \gamma \cdot \left(\sum_k a_{ik}\right) \leq 0 = V_i^\star,$$

where the contribution of the centrality and the clustering terms are null since there are no links in the network other than i's potential outgoing ties. Therefore, the necessary conditions are also sufficient for NE, i.e., the empty network is always a Nash equilibrium.

b) We then consider PNE, starting from condition (C2). Invoking Theorem 5 and recalling Remark 2, we know that the necessary condition of the Nash equilibrium which has just been discussed is equivalent to the necessary and sufficient condition for (C2). This allows to conclude that (C2) is satisfied everywhere in the parameter space.

It remains to consider the Pareto optimality condition (C3).

($\Longrightarrow$): We start by writing the utility functions of an arbitrary pair of agents (i, j):

$$\begin{aligned} V_i\left(a_{ij}, a_{ji}, \mathbf{a}_{-(i,j)}^\star\right) &= \alpha\left(a_{ji} + \delta a_{ij}a_{ji} + \delta^2 a_{ij}a_{ji}^2\right) \\ &\quad + \beta \cdot 0 - \gamma(a_{ij}) \\ V_j\left(a_{ij}, a_{ji}, \mathbf{a}_{-(i,j)}^\star\right) &= \alpha\left(a_{ij} + \delta a_{ji}a_{ij} + \delta^2 a_{ji}a_{ij}^2\right) \\ &\quad + \beta \cdot 0 - \gamma(a_{ji}). \end{aligned}$$

To derive a necessary condition, we consider the meeting strategy $(a_{ij} = 1, a_{ji} = 1)$, and we compute the respective payoff functions

$$\begin{aligned} V_i\left(1, 1, \mathbf{a}_{-(i,j)}^\star\right) &= \alpha(1 + \delta + \delta^2) - \gamma, \\ V_j\left(1, 1, \mathbf{a}_{-(i,j)}^\star\right) &= \alpha(1 + \delta + \delta^2) - \gamma. \end{aligned}$$

Note that $V_i\left(1, 1, \mathbf{a}_{-(i,j)}^\star\right) = V_j\left(1, 1, \mathbf{a}_{-(i,j)}^\star\right)$. Then, a necessary condition for (C3) to hold is certainly $V_i(1, 1, \mathbf{a}_{-(i,j)}^\star) \leq V_i^\star = 0$, otherwise the two agents can simultaneously be better off. Thus, we need the cost parameter to satisfy the following lower bound $\gamma \geq \alpha\left(1 + \delta + \delta^2\right)$.

($\Longleftarrow$): We finally prove that $\gamma \geq \alpha\left(1 + \delta + \delta^2\right)$ is a sufficient condition. For any meeting strategy (a_{ij}, a_{ji}),

$$\begin{aligned} & V_i\left(a_{ij}, a_{ji}, \mathbf{a}_{-(i,j)}^\star\right) + V_j\left(a_{ij}, a_{ji}, \mathbf{a}_{-(i,j)}^\star\right) = \\ & = \alpha\left(a_{ji} + a_{ij} + 2\delta\left(a_{ij}a_{ji}\right)\right) + \\ & + \alpha\delta^2\left(a_{ij}^2 a_{ji} + a_{ji}^2 a_{ij}\right) + \\ & - \underbrace{\gamma}_{\geq \alpha\left(1+\delta+\delta^2\right)} \left(a_{ij} + a_{ji}\right) = \\ & \leq \alpha\left(a_{ji} + a_{ij} + 2\delta\left(a_{ij}a_{ji}\right)\right) + \\ & + \alpha\delta^2\left(a_{ij}^2 a_{ji} + a_{ji}^2 a_{ij}\right) + \\ & - \alpha\left(1 + \delta + \delta^2\right)\left(a_{ij} + a_{ji}\right) = \\ & = \alpha\delta\left(2a_{ij}a_{ji} - \left(a_{ij} + a_{ji}\right)\right) + \\ & + \alpha\delta^2 \underbrace{\left(a_{ij}\left(a_{ji}^2 - 1\right) + a_{ji}\left(a_{ij}^2 - 1\right)\right)}_{\leq 0} = \\ & \leq \alpha\delta \underbrace{\left(a_{ij}\left(a_{ji} - 1\right) + a_{ij}\left(a_{ji} - 1\right)\right)}_{\leq 0} \leq 0 = \\ & = V_i^\star + V_j^\star. \end{aligned}$$

Invoking the result of Theorem 6 (ii), allows to conclude the proof.

□

Thus, we showed that the empty network is always a Nash equilibrium, conversely its pairwise-Nash stability is guaranteed only when two agents have no incentive in cooperating, i.e., creating a bilateral mutual link.

Complete Network

Next, we move to the analysis of the opposite case, the complete network. We first recall the definition, and then present the results on its equilibria conditions.

Definition (Complete Network)**.** *We call complete network (CN) a graph $\mathscr{G}^{CN}$ of N nodes such that for every pair of different agents i and j, $a_{ij} = a_{ji} = 1$.*

Theorem 8. *Let $\mathscr{G}^{CN}$ be a complete network. Define*

$$\bar{\gamma}_{NE} := \begin{cases} \alpha\delta\left(1 + \delta(2N - 3)\right) + \beta\left(N - 2\right), & \text{if } \beta > 0 \\ \alpha\delta\left(1 + \delta(2N - 3)\right) + 2\beta\left(N - 2\right), & \text{if } \beta \leq 0, \end{cases}$$

$$\bar{\gamma}_{PNE} := \alpha\delta\left(1 + \delta(2N - 3)\right) + 2\beta\left(N - 2\right), \quad \text{then}$$

a) $\mathscr{G}^{CN}$ is a NE if and only if $\gamma \leq \bar{\gamma}_{NE}$,

b) $\mathscr{G}^{CN}$ is a PNE if and only if $\gamma \leq \bar{\gamma}_{PNE}$.

Proof. We will prove the theorem in the following way. Firstly, we will discuss the NE notion, and we will derive the necessary condition for (C1) using the VI approach. Then, we will add an extra necessary condition which prevents from having agents being better off by removing all their links simultaneously. Finally, we will use these necessary conditions in order to prove

sufficiency. Secondly, we will discuss the pairwise-Nash equilibrium condition. After recalling the necessary condition derived through the Variational Inequality, we will verify that this condition is sufficient in order to guarantee both the Nash and the Pareto Optimality properties, thus the pairwise-Nash stability.

a) ($\Longrightarrow$): We start by deriving the necessary condition for NE given by the Variational Inequality [6] in Theorem 3, evaluated at the complete network. Thanks to the symmetry of the topology, we can consider, without loss of generality, any agent i, whose utility function reads as

$$\begin{aligned} V_i(\mathbf{a}_i, \mathbf{a}^\star_{-i}) = \alpha \left((N-1) + \delta \left((N-2)(N-1) + \sum_{j\neq i} a_{ij} \right) \right) + \\ + \alpha\delta^2 \left((N-1)(N-2)^2 + (2N-3) \sum_{j\neq i} a_{ij} \right) + \\ + \beta \left(\sum_{j\neq i} a_{ij} \left(\sum_{k\neq i,j} a_{ik} \right) \right) - \gamma \left(\sum_{j\neq i} a_{ij} \right). \end{aligned} \quad [11]$$

Differentiating with respect to a_{ij} and evaluating at $\mathbf{a}^\star_i$

$$\left.\frac{\partial V_i(\mathbf{a}_i, \mathbf{a}^\star_{-i})}{\partial a_{ij}}\right|_\star = \alpha\delta\left((1 + (2N-3)\right) + 2\beta\,(N-2) - \gamma$$

yields the following necessary condition from [6]: for all j different from i, for all $a_{ij} \in [0,1]$

$$\left(\alpha\delta \Big(1 + \delta\,(2N-3) \Big) + 2\beta\,(N-2) - \gamma \right) (a_{ij} - 1) \leq 0.$$

Since $(a_{ij} - 1) \leq 0$, the parametric condition reads as:

$$\gamma \leq \alpha\delta\,(1 + \delta(2N-3)) + 2\beta\,(N-2) =: \gamma_{NC}. \quad [12]$$

Note that the upper bound γ_{NC} corresponds to $\bar{\gamma}_{NE}$ for negative values of β. Conversely, for positive values of β we derive a stricter necessary condition. Consider the action $\mathbf{a}_i$ being the zero row vector (null strategy), denoted as $\mathbf{0}^T_N$, and define $\Delta = V_i(\mathbf{0}^T_N, \mathbf{a}^\star_{-i}) - V_i(\mathbf{a}^\star_i, \mathbf{a}^\star_{-i})$ as the difference between the value of the utility function at the null strategy and this at the desired equilibrium. The utility of agent i at the complete network is

$$\begin{aligned} V_i(\mathbf{a}^\star_i, \mathbf{a}^\star_{-i}) = \alpha\Big((N-1) + \delta(N-1)^2 + \delta^2(N-1)^3 \Big) + \\ + \beta(N-1)(N-2) - \gamma(N-1). \end{aligned} \quad [13]$$

By using [11], evaluated at the null strategy, one gets

$$\begin{aligned} \Delta = \alpha \Big[(N-1) + \delta(N-2)(N-1)) + \delta^2(N-1)(N-2)^2 \Big] + \\ - \Big[\alpha\Big((N-1) + \delta(N-1)^2 + \delta^2(N-1)^3 \Big) + \\ + \beta(N-1)(N-2) - \gamma(N-1) \big] = \\ = -(N-1)\Big[\alpha\delta\,(1 + \delta\,(2N-3)) + \beta\,(N-2) - \gamma \Big]. \end{aligned}$$

According to the Nash equilibrium definition, the zero-vector cannot be a better strategy for i, therefore we must have $\Delta \leq 0$. This yields to the following condition

$$\gamma \leq \alpha\delta\,(1 + \delta\,(2N-3)) + \beta\,(N-2) := \gamma_{NC,2}. \quad [14]$$

Comparing [12] and [14] one notes that $\gamma_{NC} \leq \gamma_{NC,2}$ for negative values of β, and vice versa for positive values of β. Combining the two bounds, we obtain that the necessary condition is $\gamma \leq \bar{\gamma}_{NE}$.

($\Longleftarrow$) In order to check sufficiency, we assume $\gamma \leq \bar{\gamma}_{NE}$ and we prove the NE condition. In the following, we compute $\Delta(\mathbf{a}_i) = V_i(\mathbf{a}_i, \mathbf{a}^\star_{-i}) - V_i(\mathbf{a}^\star_i, \mathbf{a}^\star_{-i})$ using [11] and [13], and we show that it is always non-positive.

$$\begin{aligned} \Delta(\mathbf{a}_i) = \alpha\delta\,(1 + (2N-3)\,\delta) \left(\sum_{j\neq i} a_{ij} - (N-1) \right) + \\ + \beta \left(\sum_{j\neq i} a_{ij} \left(\sum_{k\neq i,j} a_{ik} \right) - (N-1)(N-2) \right) + \\ + \gamma \underbrace{\left(N - 1 - \sum_{j\neq i} a_{ij} \right)}_{(*)} \end{aligned}$$

We need to distinguish two cases: first, consider $\beta \geq 0$, then the necessary condition $\gamma \leq \bar{\gamma}_{NE}$ reads as $\gamma \leq \alpha\delta\,(1 + \delta(2N-3)) + \beta\,(N-2)$. Since $(*) \geq 0$, we can use the upper bound $\bar{\gamma}_{NE}$ and get

$$\begin{aligned} \Delta(\mathbf{a}_i) \leq \cancel{\alpha\delta\,(1 + \delta\,(2N-3)) \left(\sum_{j\neq i} a_{ij} - (N-1) \right)} + \\ + \beta \left(\sum_{j\neq i} a_{ij} \left(\sum_{k\neq i,j} a_{ik} \right) - (N-1)(N-2) \right) + \\ + \cancel{\alpha\delta\,(1 + \delta\,(2N-3)) \left(N - 1 - \sum_{j\neq i} a_{ij} \right)} + \\ + \beta\,(N-2) \left(N - 1 - \sum_{j\neq i} a_{ij} \right) = \\ = \beta \left(\sum_{j\neq i} a_{ij} \left(\sum_{k\neq i,j} a_{ik} \right) - (N-2) \sum_{j\neq i} a_{ij} \right) = \\ = \underbrace{\beta}_{\geq 0} \left(\sum_{j\neq i} a_{ij} \underbrace{\left(\sum_{k\neq i,j} a_{ik} - (N-2) \right)}_{\leq 0} \right) \leq 0. \end{aligned}$$

Vice versa, if $\beta < 0$, then the necessary condition is

$\gamma \le \alpha\delta(1+\delta(2N-3)) + 2\beta(N-2)$ and

$$\begin{aligned}\Delta(\mathbf{a}_i) \le\; & \cancel{\alpha\delta(1+\delta(2N-3))}\left(\sum_{j\neq i} a_{ij} - (N-1)\right) + \\ & + \beta\left(\sum_{j\neq i} a_{ij}\left(\sum_{k\neq i,j} a_{ik}\right) - (N-1)(N-2)\right) + \\ & + \cancel{\alpha\delta(1+\delta(2N-3))}\left(N-1-\sum_{j\neq i} a_{ij}\right) + \\ & + 2\beta(N-2)\left(N-1-\sum_{j\neq i} a_{ij}\right) = \\ & = \beta f(\mathbf{a}_i),\end{aligned} \quad [15]$$

where we used the shorthand

$$f(\mathbf{a}_i) = \sum_{j\neq i} a_{ij}\left(\sum_{k\neq i,j} a_{ik} - 2(N-2)\right) + (N-2)(N-1).$$

Consider $f(\mathbf{a}_i)$ and compute for all j different from i,

$$\frac{\partial f(\mathbf{a}_i)}{\partial a_{ij}} = -2\left(N-2-\sum_{k\neq i,j} a_{ik}\right) \le 0, \quad \forall \mathbf{a}_i \in \mathscr{A},$$

therefore the minimum of $f(\mathbf{a}_i)$ is obtained when $a_{ij} = 1$ for all $j \neq i$, leading to $f(\mathbf{a}_i) \ge f(\mathbf{a}_i^\star) = 0, \forall \mathbf{a}_i \in \mathscr{A}$. Thus, since $\beta < 0$ in this case, from [15] it follows $\Delta(\mathbf{a}_i) \le 0$ for all $\mathbf{a}_i \in \mathscr{A}$, and this completes the proof of the Nash equilibrium conditions.

b) According to the result in Theorem 5 and recalling Remark 2, the necessary and sufficient condition for satisfying the NE property (C2) of the PNE-stability definition is exactly $\gamma \le \gamma_{NC} = \bar{\gamma}_{PNE}$, as derived in [12].

($\Longleftarrow$): It remains to show that $\gamma \le \bar{\gamma}_{PNE}$ is sufficient in order to satisfy the Pareto-optimality condition (C3). However, given that for any pair (i,j), the equilibrium strategy is precisely $(a_{ij}^\star, a_{ji}^\star) = (1,1)$, we can invoke Theorem 6 (i) and immediately conclude.

□

Even though the stability conditions of the complete network topology are different for the two equilibrium notions, the discrepancy is noticeable only when zooming close to the origin, as shown in Supplementary Fig. 2.

Balanced Complete Bipartite Network

After the analysis of the two extreme cases, empty and complete network topologies, we present the results on the equilibrium of two types of bipartite networks. We recall that these are networks in which nodes can be partitioned into two subsets such that ties only exist between nodes belonging to the two different factions. The balanced complete bipartite network belongs to this type of topologies and it features the following properties: it is balanced, i.e., the number of nodes is evenly distributed in the two factions, and it is complete, i.e., all the pair of nodes belonging to the two different factions have reciprocated ties of weight one. Hereafter we recall the definition, and afterwards we present the result in the following theorem.

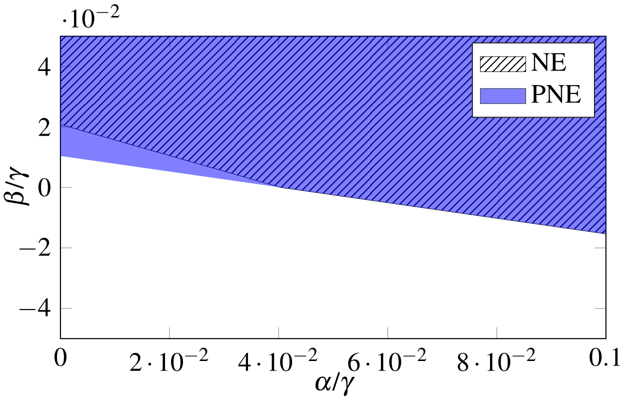

Supplementary Figure 2. Particular of the stability region of the complete network, for $N = 50$ and $\delta = 0.5$.

Definition (Balanced Complete Bipartite Network)**.** *We call balanced complete bipartite network (BN) a graph $\mathscr{G}^{BN}$ of N nodes (with N being even) which can be partitioned into two subsets of $N/2$ agents each, such that $a_{ij} = 1$ for all pairs (i,j) of agents belonging to different factions, and $a_{ij} = 0$ otherwise.*

Theorem 9. *Let $\mathscr{G}^{BN}$ be a balanced complete bipartite network of N agents, with N being even. Define*

$$\begin{aligned}\underline{\gamma}_{NE} &:= \alpha\delta^2\left(\frac{N}{2}\right) + 2\beta\left(\frac{N}{2}\right), \\ \underline{\gamma}_{PNE} &:= \alpha\left(1+\delta\left(\frac{N}{2}+1\right)+\delta^2\left(\left(\frac{N}{2}\right)^2+3\frac{N}{2}+1\right)\right)+2\beta\left(\frac{N}{2}\right), \\ \bar{\gamma}_{NE} = \bar{\gamma}_{PNE} &:= \alpha\delta\left(1+\delta\frac{N}{2}\right),\end{aligned}$$

then

a) $\mathscr{G}^{BN}$ is a NE if and only if $\underline{\gamma}_{NE} \le \gamma \le \bar{\gamma}_{NE}$,

b) $\mathscr{G}^{BN}$ is a PNE if and only if $\underline{\gamma}_{PNE} \le \gamma \le \bar{\gamma}_{PNE}$.

Proof. a) ($\Longrightarrow$) : As in the previous proof, we start by deriving the NE necessary condition with the VI approach. As the network is balanced bipartite, nodes can be split into two groups. Without loss of generality, let them being $V_1 = \{1,\ldots,N/2\}$ and $V_2 = \{N/2+1,\ldots,N\}$. Given the symmetry of the topology, we consider only $i \in V_1$, without loss of generality. The payoff function for a general action

$\mathbf{a}_i$ is as follows

$$
\begin{aligned}
&V_i(\mathbf{a}_i,\mathbf{a}^\star_{-i}) = \\
&\Bigg[\alpha\cdot\left(\sum_{k\neq i} a_{ki} + \delta\sum_{l\neq k}\sum_{k\neq i} a_{lk}a_{ki} + \right.\\
&+\left.\left(\delta^2\sum_{m\neq l}\sum_{l\neq k}\sum_{k\neq i} a_{ml}a_{lk}a_{ki}\right)\right) + \\
&+\beta\cdot\left(\sum_{k\neq i} a_{ik}\left(\sum_{l\neq i,k} a_{il}a_{lk}\right)\right) - \gamma\cdot\left(\sum_{k\neq i} a_{ik}\right)\Bigg|_{(\mathbf{a}_i,\mathbf{a}^\star_{-i})}.
\end{aligned}
$$

By applying the value of $\mathbf{a}^\star_{-i}$ and isolating the contribution of $a_{i\bullet}$, we obtain

$$
\begin{aligned}
&V_i(\mathbf{a}_i,\mathbf{a}^\star_{-i}) = \\
&=\alpha\left(\sum_{\substack{k\in V_1\\k\neq i}} \underbrace{a_{ki}}_{=0} + \sum_{k\in V_2}\underbrace{a_{ki}}_{=1}\right) + \\
&+\alpha\delta\left(\sum_{l\neq k}\sum_{\substack{k\in V_1\\k\neq i}} a_{lk}\underbrace{a_{ki}}_{=0} + \sum_{\substack{l\in V_2\\l\neq k}}\sum_{k\in V_2}\underbrace{a_{lk}}_{=0}a_{ki}\right) + \\
&+\alpha\delta\left(\sum_{\substack{l\in V_1\\l\neq i}}\sum_{k\in V_2}\underbrace{a_{lk}}_{=1}\underbrace{a_{ki}}_{=1} + \sum_{k\in V_2}\underbrace{a_{ki}}_{=1}a_{ik}\right) + \\
&+\alpha\delta^2\left(\sum_{m\neq l}\sum_{l\neq k}\sum_{\substack{k\in V_1\\k\neq i}} a_{ml}a_{lk}\underbrace{a_{ki}}_{=0} + \sum_{m\neq l}\sum_{\substack{l\in V_2\\l\neq k}}\sum_{k\in V_2} a_{ml}\underbrace{a_{lk}}_{=0}a_{ki}\right) + \\
&+\alpha\delta^2\left(\sum_{\substack{m\in V_1\\m\neq l}}\sum_{\substack{l\in V_1\\l\neq i}}\sum_{k\in V_2}\underbrace{a_{ml}}_{=0}a_{lk}a_{ki} + \sum_{\substack{l\in V_1\\l\neq i}}\sum_{k\in V_2} a_{il}\underbrace{a_{lk}}_{=1}\underbrace{a_{ki}}_{=1}\right) + \\
&+\alpha\delta^2\left(\sum_{m\in V_2}\sum_{\substack{l\in V_1\\l\neq i}}\sum_{k\in V_2}\underbrace{a_{ml}}_{=1}\underbrace{a_{lk}}_{=1}\underbrace{a_{ki}}_{=1} + \sum_{m\in V_2}\sum_{k\in V_2}\underbrace{a_{mi}}_{=1}a_{ik}\underbrace{a_{ki}}_{=1}\right) + \\
&+\beta\left(\sum_{k\in V_2} a_{ik}\left(\sum_{l\in V_2} a_{il}\underbrace{a_{lk}}_{=0}\right) + \sum_{\substack{k\in V_1\\k\neq i}} a_{ik}\left(\sum_{\substack{l\in V_1\\l\neq i}} a_{il}\underbrace{a_{lk}}_{=0}\right)\right) + \\
&+\beta\left(\sum_{\substack{k\in V_1\\k\neq i}} a_{ik}\left(\sum_{l\in V_2} a_{il}\underbrace{a_{lk}}_{=1}\right) + \sum_{k\in V_2} a_{ik}\left(\sum_{\substack{l\in V_1\\l\neq i}} a_{il}\underbrace{a_{lk}}_{=1}\right)\right) + \\
&-\gamma\cdot\left(\sum_{k\neq i} a_{ik}\right) =
\end{aligned}
$$

$$
\begin{aligned}
&=\alpha\left(\frac{N}{2} + \delta\left(\left(\frac{N}{2}\right)\left(\frac{N}{2}-1\right) + \sum_{k\in V_2} a_{ik}\right)\right) + \\
&+\alpha\delta^2\left(\left(\frac{N}{2}\right)^2\left(\frac{N}{2}-1\right) + \frac{N}{2}\left(\sum_{k\in V_2} a_{ik} + \sum_{\substack{k\in V_1\\k\neq i}} a_{ik}\right)\right) + \\
&+2\beta\left(\sum_{\substack{k\in V_1\\k\neq i}} a_{ik}\sum_{l\in V_2} a_{il}\right) - \gamma\left(\sum_{k\neq i} a_{ik}\right).
\end{aligned}
$$

The gradient of V_i at the equilibrium strategy is then

$$
\left.\frac{\partial V_i(\mathbf{a}_i,\mathbf{a}^\star_{-i})}{\partial a_{ij}}\right|_\star = \begin{cases}\alpha\delta^2\left(\frac{N}{2}\right) + 2\beta\left(\frac{N}{2}\right) - \gamma, & j\in V_1, j\neq i,\\ \alpha\delta\left(1+\delta\frac{N}{2}\right) - \gamma, & j\in V_2.\end{cases}
$$

Applying the Variational Inequality [6] in Theorem 3

$$
\begin{aligned}
&\left(\alpha\delta^2\left(\frac{N}{2}\right) + 2\beta\left(\frac{N}{2}\right) - \gamma\right)\cdot(a_{ij}-0)\leq 0, \forall j\neq i\in V_1,\ \forall a_{ij}\in[0,1],\\
&\left(\alpha\delta\left(1+\delta\frac{N}{2}\right) - \gamma\right)\cdot(a_{ij}-1)\leq 0, \forall j\in V_2,\ \forall a_{ij}\in[0,1],
\end{aligned}
$$

yields the following parametric conditions

$$
\begin{aligned}
\alpha\delta^2\left(\frac{N}{2}\right) + 2\beta\left(\frac{N}{2}\right) - \gamma &\leq 0,\\
\alpha\delta\left(1+\delta\frac{N}{2}\right) - \gamma &\geq 0.
\end{aligned}
$$

Therefore, we proved that the necessary conditions are precisely

$$
\begin{aligned}
\underline{\gamma}_{NE} &:= \alpha\delta^2\left(\frac{N}{2}\right) + 2\beta\left(\frac{N}{2}\right) \leq \gamma\\
\bar{\gamma}_{NE} &:= \alpha\delta\left(1+\delta\frac{N}{2}\right) \geq \gamma.
\end{aligned} \qquad [16]
$$

($\Longleftarrow$) : In order to prove sufficiency, we first notice that the payoff function of a generic agent i depends only on two parameters. Introducing the shorthand notation

$$
x = \sum_{k\in V_2} a_{ik} \in [0, N/2], \quad y = \sum_{\substack{k\in V_1\\k\neq i}} a_{ik} \in [0, N/2-1],
$$

we can rewrite the utility function in a more compact form:

$$
\begin{aligned}
V_i(x,y,\mathbf{a}^\star_{-i}) &= \alpha\left(\frac{N}{2} + \delta\left(\left(\frac{N}{2}\right)^2 - \left(\frac{N}{2}-x\right)\right)\right) + \\
&+\alpha\delta^2\left(\left(\frac{N}{2}\right)^3 - \left(\frac{N}{2}\right)\left(\frac{N}{2}-(x+y)\right)\right) + \\
&+2\beta\,(xy) - \gamma(x+y).
\end{aligned}
$$

We then assume $\underline{\gamma}_{NE} \le \gamma \le \bar{\gamma}_{NE}$, and we compute $\Delta(x,y) = V_i(x,y,\mathbf{a}^\star_{-i}) - V_i^\star$, where

$$V_i^\star := V_i(x = N/2, y = 0, \mathbf{a}^\star_{-i}) = \\ = \alpha\left(\frac{N}{2} + \delta\left(\frac{N}{2}\right)^2 + \delta^2\left(\frac{N}{2}\right)^3\right) - \gamma\left(\frac{N}{2}\right). \quad [17]$$

In what follows, we verify that, for all $x \in [0, N/2]$, for all $y \in [0, N/2-1]$, $\Delta(x,y) \le 0$. We have

$$\Delta(x,y) = -\alpha\delta\left(\left(\frac{N}{2} - x\right) + \delta^2\left(\frac{N}{2}\right)\left(\frac{N}{2} - (x+y)\right)\right) + \\ + 2\beta\,(xy) + \gamma\left(\left(\frac{N}{2}\right) - (x+y)\right).$$

Using the first necessary condition from [16] $2\beta \le -\alpha\delta^2 + \gamma(N/2)^{-1}$, we obtain

$$\Delta(x,y) \le -\alpha\delta\left(\left(\frac{N}{2} - x\right) + \delta^2\left(\frac{N}{2}\right)\left(\frac{N}{2} - (x+y)\right)\right) + \\ - \alpha\delta^2\,(xy) + \gamma(N/2)^{-1}\,(xy) + \gamma\left(\frac{N}{2} - (x+y)\right).$$

Grouping few terms,

$$\Delta(x,y) \le -\alpha\delta\left(\frac{N}{2} - x\right) - \alpha\delta^2\left(\frac{N}{2} - x\right)\left(\frac{N}{2} - y\right) + \\ + \gamma(N/2)^{-1}\left(\frac{N}{2} - x\right)\left(\frac{N}{2} - y\right),$$

and using the other necessary condition form [16] , namely $\gamma(N/2)^{-1} \le \alpha\delta^2 + \alpha\delta\,(N/2)^{-1}$, we reach

$$\Delta(x,y) \le -\alpha\delta\left(\frac{N}{2} - x\right) - \cancel{\alpha\delta^2\left(\frac{N}{2} - x\right)\left(\frac{N}{2} - y\right)} + \\ + \cancel{\alpha\delta^2\left(\frac{N}{2} - x\right)\left(\frac{N}{2} - y\right)} + \\ + \alpha\delta\left(\frac{N}{2}\right)^{-1}\left(\frac{N}{2} - x\right)\left(\frac{N}{2} - y\right),$$

and we finally prove that, for all admissible (x,y),

$$\Delta(x,y) \le \alpha\delta\left(\frac{N}{2}\right)^{-1}\left(\frac{N}{2} - x\right)\left(\cancel{\frac{N}{2}} - y - \cancel{\frac{N}{2}}\right) \le 0,$$

which concludes the proof of the Nash equilibrium conditions.

b) $(\Longrightarrow)$: According to the result in Theorem 5 and recalling Remark 2, the necessary and sufficient condition for satisfying the NE property (C2) of the PNE-stability definition is exactly $\underline{\gamma}_{NE} \le \gamma \le \bar{\gamma}_{NE}$, as derived in [16]. For this topology, we need to introduce an extra necessary condition in order to prevent that cooperation between agents in the same group could lead to the formation of favorable mutual ties. In order to formally derive this condition, consider two distinct agents i, j in the same group, say V_1, without loss of generality. At the equilibrium topology, these agents are not linked and agent i's payoff as function of the meeting strategy (a_{ij}, a_{ji}) reads as:

$$V_i\left(a_{ij}, a_{ji}, \mathbf{a}^\star_{-(i,j)}\right) = \\ + \alpha\left(\frac{N}{2} + \delta\left(\frac{N}{2}\right)^2 + \delta^2\left(\frac{N}{2}\right)^3 + a_{ji} + \delta\left(\frac{N}{2}a_{ji} + a_{ij}a_{ji}\right)\right) + \\ + \alpha\delta^2\left(\left(\frac{N}{2}\right)^2 a_{ji} + \left(\frac{N}{2}\right)(a_{ij} + a_{ji} + a_{ij}a_{ji}) + a_{ij}a_{ji}^2\right) + \\ + 2\beta\left(\frac{N}{2}\right)a_{ij} - \gamma\left(\frac{N}{2} + a_{ij}\right). \quad [18]$$

Thanks to the symmetry of the topology, the payoff function of agent j is analogous. Consider the mutual strategy $(1,1)$, then the Pareto optimality condition (C3) requires that $\Delta = V_i\left(1, 1, \mathbf{a}^\star_{-(i,j)}\right) - V_i^\star \le 0$, where $V_i^\star = V_i\left(0, 0, \mathbf{a}^\star_{-(i,j)}\right)$ was previously derived in [17]. For otherwise, both agents would benefit from switching from the current un-linked situation in favor of being mutually linked, violating (C3). Then, imposing

$$\Delta = \alpha\left(1 + \delta\left(\frac{N}{2} + 1\right) + \delta^2\left(\left(\frac{N}{2}\right)^2 + 3\left(\frac{N}{2}\right) + 1\right)\right) + \\ + 2\beta\left(\frac{N}{2}\right) - \gamma \le 0,$$

leads exactly to the necessary condition $\gamma \ge \underline{\gamma}_{PNE}$ as in the statement of the theorem, namely

$$\gamma \ge \alpha\left(1 + \delta\left(\frac{N}{2} + 1\right) + \delta^2\left(\left(\frac{N}{2}\right)^2 + 3\left(\frac{N}{2}\right) + 1\right)\right) + \\ + 2\beta\left(\frac{N}{2}\right) = \underline{\gamma}_{PNE}. \quad [19]$$

One can notice that this condition is actually more restrictive than the NE condition [16], i.e., $\underline{\gamma}_{PNE} \ge \underline{\gamma}_{NE}$. This becomes obvious from the direct comparison of the α coefficients of the two lower bounds

$$\underline{\gamma}_{NE} = \alpha\delta^2\left(\frac{N}{2}\right) + 2\beta\left(\frac{N}{2}\right)$$
$$\underline{\gamma}_{PNE} = \alpha\left(1 + \delta\,(N/2+1)\right) + \alpha\delta^2\left(\left(\frac{N}{2}\right)^2 + 3\frac{N}{2} + 1\right) + 2\beta\frac{N}{2}.$$

$(\Longleftarrow)$: We finally prove sufficiency of these conditions. We already know that the necessary condition is also a sufficient condition for the Nash property (C2). It therefore remains to verify the Pareto optimality condition. Given the symmetry of the topology, it is sufficient to pick

- the pair (i,j) when $i \in V_1$ and $j \in V_2$,

- the pair (i,j) when $i \in V_1$ and $j \in V_1$, with $j \neq i$.

However, for any pair (i,j) of the first type the equilibrium strategy is precisely $(a^\star_{ij}, a^\star_{ji}) = (1,1)$. Therefore invoking Theorem 6 (i) allows to conclude that condition (C3) is satisfied for these pairs. Thus, it remains to consider a pair of agents in the same group. We have already derived in [18] the expression of the utility function of agent i in this case, and for agent j we have an analogous formulation. Consider

$$\begin{aligned}
&\Delta(a_{ij}, a_{ji}) = \\
&V_i\left(a_{ij}, a_{ji}, \mathbf{a}^\star_{-(i,j)}\right) + V_j\left(a_{ij}, a_{ji}, \mathbf{a}^\star_{-(i,j)}\right) - \left(V_i^\star + V_j^\star\right) = \\
&= \alpha\left(\left(a_{ij}+a_{ji}\right) + \delta\left(\frac{N}{2}\left(a_{ij}+a_{ji}\right) + 2a_{ij}a_{ji}\right)\right) + \\
&+ \alpha\delta^2\left(\left(\frac{N}{2}\right)^2\left(a_{ij}+a_{ji}\right) + 2\frac{N}{2}\left(a_{ij}+a_{ji}+a_{ij}a_{ji}\right)\right) + \\
&+ \alpha\delta^2 a_{ij}a_{ji}\left(a_{ij}+a_{ji}\right) + 2\beta\frac{N}{2}\left(a_{ij}+a_{ji}\right) - \gamma\left(a_{ij}+a_{ji}\right).
\end{aligned}$$

Using the necessary condition derived in [19], namely

$$2\beta\left(\frac{N}{2}\right) - \gamma \leq -\alpha\left(1+\delta\left(\frac{N}{2}+1\right) + \delta^2\left(\left(\frac{N}{2}\right)^2 + 3\left(\frac{N}{2}\right) + 1\right)\right),$$

we obtain that $\Delta(a_{ij}, a_{ji})$ is upper-bounded by

$$\begin{aligned}
&\leq \alpha\left(\cancel{\left(a_{ij}+a_{ji}\right)} + \delta\left(\cancel{\frac{N}{2}\left(a_{ij}+a_{ji}\right)} + 2a_{ij}a_{ji}\right)\right) + \\
&+ \alpha\delta^2\left(\cancel{\left(\frac{N}{2}\right)^2\left(a_{ij}+a_{ji}\right)} + 2\frac{N}{2}\left(a_{ij}+a_{ji}+a_{ij}a_{ji}\right)\right) + \\
&+ \alpha\delta^2 a_{ij}a_{ji}\left(a_{ij}+a_{ji}\right) - \alpha\left(\cancel{1}+\delta\left(\cancel{\frac{N}{2}}+1\right)\right)\left(a_{ij}+a_{ji}\right) + \\
&- \alpha\delta^2\left(\cancel{\left(\frac{N}{2}\right)^2} + 3\left(\frac{N}{2}\right) + 1\right)\left(a_{ij}+a_{ji}\right) = \\
&= \alpha\delta\left(2a_{ij}a_{ji} - a_{ij} - a_{ji}\right) + \\
&+ \alpha\delta^2\frac{N}{2}\left(2a_{ij} + 2a_{ji} + 2a_{ij}a_{ji} - 3a_{ij} - 3a_{ji}\right) + \\
&+ \alpha\delta^2\left(a_{ij}+a_{ji}\right)\left(a_{ij}a_{ji} - 1\right) = \\
&= \underbrace{-\alpha\delta\left(1+\delta\frac{N}{2}\right)\left(a_{ij}\left(1-a_{ji}\right) + a_{ji}\left(1-a_{ij}\right)\right)}_{\leq 0} + \\
&\underbrace{-\alpha\delta^2\left(a_{ij}+a_{ji}\right)\left(1-a_{ij}a_{ji}\right)}_{\leq 0} \leq 0.
\end{aligned}$$

Thus, we also showed that for all pairs (i,j) and for all pairs (a_{ij}, a_{ji}) in $[0,1]^2$,

$$V_i\left(a_{ij}, a_{ji}, \mathbf{a}^\star_{-(i,j)}\right) + V_j\left(a_{ij}, a_{ji}, \mathbf{a}^\star_{-(i,j)}\right) \leq V_i^\star + V_j^\star.$$

Invoking Theorem 6 (ii) allows to conclude the proof.

□

Star Network

Lastly, we consider the star network topology, a very unbalanced example of complete bipartite network. In this case there are two types of nodes: the central node and the periphery nodes, defining the two partitions. As in the balanced complete bipartite network, the edges of the star network consist of all the possible ties between two nodes in the two different partitions.

Definition (Star Network)**.** *We call star network (SN) a graph $\mathscr{G}^{SN}$ of N nodes such that there exists an agent r, called the center of the star, such that $a_{rj} = a_{jr} = 1$ for all other agents j, while for all the other links a_{ij} in the network, $a_{ij} = 0$.*

Theorem 10. *Let $\mathscr{G}^{SN}$ be a star network. Define*

$$\begin{aligned}
\underline{\gamma}_{NE} &:= \max\left\{\alpha\delta^2 + 2\beta, \alpha\delta\left(\delta - \frac{1}{N-3}\right)\right\}, \\
\underline{\gamma}_{PNE} &:= \alpha\left(1 + 2\delta + (N+3)\delta^2\right) + 2\beta, \\
\bar{\gamma}_{NE} &= \bar{\gamma}_{PNE} := \alpha\delta\left(1+\delta\right),
\end{aligned}$$

then

a) $\mathscr{G}^{SN}$ is a NE if and only if $\underline{\gamma}_{NE} \leq \gamma \leq \bar{\gamma}_{NE}$,

b) $\mathscr{G}^{SN}$ is a PNE if and only if $\underline{\gamma}_{PNE} \leq \gamma \leq \bar{\gamma}_{PNE}$.

Proof. a) $(\Longrightarrow)$: As in the previous proofs, we start by deriving the NE necessary conditions with the VI approach. Firstly, we explicitly derive the actions at the star network, assuming, without loss of generality, that the node 1 is the center of the star

$$\mathbf{a}_1^\star = \begin{bmatrix} 0 & \mathbf{1}^T_{N-1,1} \end{bmatrix}, \quad \mathbf{a}_i^\star = \begin{bmatrix} 1, & \mathbf{0}^T_{N-1,1} \end{bmatrix}, \forall i \neq 1.$$

Note that thanks to the symmetry of the topology, we can simply consider two types of nodes namely, the central node 1 and a generic peripheral node $i \neq 1$. Therefore, in the following, we always refer to i as a generic node in the periphery of the star. We then derive the expression for the payoff functions of the two types of nodes. For the center of the star we obtain

$$\begin{aligned}
&V_1(\mathbf{a}_1, \mathbf{a}^\star_{-1}) = \\
&= \Bigg[\alpha\left(\sum_{k\neq 1} a_{k1} + \delta\sum_{l\neq k}\sum_{k\neq 1} a_{lk}a_{k1} + \right) + \\
&+ \alpha\delta^2\sum_{m\neq l}\sum_{l\neq k}\sum_{k\neq 1} a_{ml}a_{lk}a_{k1} + \\
&+ \beta\left(\sum_{k\neq 1} a_{1k}\left(\sum_{l\neq 1,k} a_{1l}a_{lk}\right)\right) - \gamma\left(\sum_{k\neq 1} a_{1k}\right)\Bigg|_{(\mathbf{a}_1, \mathbf{a}^\star_{-1})}.
\end{aligned}$$

By applying the value of $\mathbf{a}^\star_{-1}$ and isolating the contribution of $a_{1\bullet}$,

$$
\begin{aligned}
V_1(\mathbf{a}_1,\mathbf{a}^\star_{-1}) &= \alpha\,(N-1) + \alpha\delta \left(\cancel{\sum_{l\neq k,1}\sum_{k\neq 1} \underbrace{a_{lk}}_{=0} a_{k1}} + \sum_{k\neq 1} a_{1k} \underbrace{a_{k1}}_{=1} \right) + \\
&+ \alpha\delta^2 \left(\cancel{\sum_{m\neq l,1}\sum_{l\neq k,1}\sum_{k\neq 1} a_{ml} \underbrace{a_{lk}}_{=0} a_{k1}} + \cancel{\sum_{l\neq k,1}\sum_{k\neq 1} a_{1l} \underbrace{a_{lk}}_{=0} a_{k1}} \right) + \\
&+ \alpha\delta^2 \left(\sum_{m\neq 1}\sum_{k\neq 1} \underbrace{a_{m1}}_{=1} a_{1k} \underbrace{a_{k1}}_{=1} \right) + \\
&+ \beta \left(\cancel{\sum_{k\neq 1} a_{1k} \left(\sum_{l\neq 1,k} a_{1l} \underbrace{a_{lk}}_{=0} \right)} \right) - \gamma \left(\sum_{k\neq 1} a_{1k} \right) = \\
&= \alpha \left((N-1) + \delta \left(\sum_{k\neq 1} a_{1k} \right) + \delta^2\,(N-1) \left(\sum_{k\neq 1} a_{1k} \right) \right) + \\
&- \gamma \left(\sum_{k\neq 1} a_{1k} \right).
\end{aligned}
\tag{20}
$$

Evaluating the payoff function for the central node, at the star network topology, we obtain

$$
\begin{aligned}
V_1^\star &:= V_1(\mathbf{a}_1^\star,\mathbf{a}^\star_{-1}) \\
&= \alpha\left((N-1) + \delta\,(N-1) + \delta^2\,(N-1)\ \right) - \gamma(N-1).
\end{aligned}
$$

Conversely, for a peripheral node $i \neq 1$ we get

$$
\begin{aligned}
V_i(\mathbf{a}_i,\mathbf{a}^\star_{-i}) = \Bigg[&\alpha \cdot \left(\sum_{k\neq i} a_{ki} + \delta \sum_{l\neq k}\sum_{k\neq i} a_{lk}a_{ki} \right) + \\
&+ \alpha\delta^2 \sum_{m\neq l}\sum_{l\neq k}\sum_{k\neq i} a_{ml}a_{lk}a_{ki} \\
&+ \beta \left(\sum_{k\neq i} a_{ik} \left(\sum_{l\neq i,k} a_{il}a_{lk} \right) \right) - \gamma \left(\sum_{k\neq i} a_{ik} \right) \Bigg|_{(\mathbf{a}_i,\mathbf{a}^\star_{-i})}.
\end{aligned}
$$

Given the particular topology, we highlight the contribution deriving from the elements $a_{\bullet 1}$ and $a_{1\bullet}$, as they are the only non-null elements of $\mathbf{a}^\star_{-i}$.

$$
\begin{aligned}
&V_i(\mathbf{a}_i,\mathbf{a}^\star_{-i}) = \\
&= \Bigg[\alpha \left(\sum_{k\neq i,1} \cancel{a_{ki}} + a_{1i} \right) + \alpha\delta \left(\sum_{l\neq k}\sum_{k\neq i,1} a_{lk}\cancel{a_{ki}} \right) + \\
&+ \alpha\delta \left(\sum_{l\neq i,1} a_{l1}a_{1i} + a_{i1}a_{1i} \right) + \alpha\delta^2 \left(\sum_{m\neq l}\sum_{l\neq k}\sum_{k\neq i,1} a_{ml}a_{lk}\cancel{a_{ki}} \right) + \\
&+ \alpha\delta^2 \left(\sum_{m\neq l,1,i}\sum_{l\neq i} \cancel{a_{ml}} a_{l1}a_{1i} + \sum_{l\neq 1,i} a_{il}a_{l1}a_{1i} \right) + \\
&+ \alpha\delta^2 \left(\sum_{l\neq 1,i} a_{1l}a_{l1}a_{1i} + a_{1i}a_{i1}a_{1i} \right) + \\
&+ \beta \left(\sum_{k\neq i,1} a_{ik} \left(\sum_{l\neq i,k,1} a_{il}\cancel{a_{lk}} + a_{i1}a_{1k} \right) + a_{i1} \left(\sum_{l\neq i,1} a_{il}a_{l1} \right) \right) + \\
&- \gamma \left(\sum_{k\neq i} a_{ik} \right) \Bigg|_{(\mathbf{a}_i,\mathbf{a}^\star_{-i})}.
\end{aligned}
$$

By isolating the contribution of $a_{i\bullet}$ and applying the value of $\mathbf{a}^\star_{-i}$

$$
\begin{aligned}
&V_i(\mathbf{a}_i,\mathbf{a}^\star_{-i}) = \\
&= \alpha \left(1 + \delta\,(N-2+a_{i1}) + \delta^2 \left(\sum_{l\neq 1,i} a_{il} + N - 2 + a_{i1} \right) \right) + \\
&+ 2\beta a_{i1} \sum_{k\neq i,1} a_{ik} - \gamma \cdot \left(\sum_{k\neq i} a_{ik} \right).
\end{aligned}
$$

Note that the previous expression only depends on two variables: a_{i1}, the link towards the center of the star, and $\sum_{k\neq i,1} a_{ik}$, the sum of the weights of the links towards the other peripheral nodes. Introducing the notation $x = a_{i1} \in [0,1]$ and $y = \sum_{k\neq i,1} a_{ik} \in [0, N-2]$, we obtain

$$
\begin{aligned}
V_i(x,y,\mathbf{a}^\star_{-i}) = \alpha\left(1 + \delta\,(N-2+x) + \delta^2\,(y+N-2+x)\right) + \\
+ 2\beta xy - \gamma(x+y).
\end{aligned}
\tag{21}
$$

Finally, the value of the payoff function for the peripheral node in the star network topology, i.e., when $x = 1$ and $y = 0$, results in

$$
V_i^\star := V_i(\mathbf{a}_i^\star,\mathbf{a}^\star_{-i}) = \alpha\left(1 + \delta\,(N-1) + \delta^2\,(N-1)\right) - \gamma.
\tag{22}
$$

In order to apply Theorem 2, we derive the gradient of the payoff function for the central agent

$$
\frac{\partial V_1(\mathbf{a}_1^\star,\mathbf{a}^\star_{-1})}{\partial a_{1j}} = \alpha\delta\,(1+\delta\,(N-1)) - \gamma, \qquad \forall j \neq 1,
$$

and for the generic peripheral node $i \neq 1$

$$
\frac{\partial V_i(\mathbf{a}_i^\star,\mathbf{a}^\star_{-i})}{\partial a_{ij}} = \begin{cases} \alpha\delta\,(1+\delta) - \gamma, & j = 1 \\ \alpha\delta^2 + 2\beta - \gamma, & \text{otherwise} \end{cases}
$$

Thus, applying Theorem 3,

$$
\begin{aligned}
&\left(\alpha\delta\,(1+\delta\,(N-1)) - \gamma\right)\left(a_{1j} - 1\right) \leq 0, && \forall j \neq 1, \\
& && \forall a_{1j} \in [0,1] \\
&\left(\alpha\delta\,(1+\delta) - \gamma\right)(a_{i1} - 1) \leq 0, && \forall i \neq 1, \\
& && \forall a_{i1} \in [0,1] \\
&\left(\alpha\delta^2 + 2\beta - \gamma\right)(a_{ij} - 0) \leq 0, && \forall i \neq 1, \forall j \neq i,1 \\
& && \forall a_{ij} \in [0,1]
\end{aligned}
$$

we obtain the following parametric necessary conditions

$$
\begin{cases}
\gamma & \leq \alpha\delta\,(1+\delta\,(N-1)) \\
\gamma & \leq \alpha\delta\,(1+\delta) \\
\gamma & \geq \alpha\delta^2 + 2\beta.
\end{cases}
$$

These can be combined to the reduced conditions

$$\alpha\delta^2 + 2\beta \leq \gamma \leq \alpha\delta(1+\delta) = \bar{\gamma}_{NE}. \qquad [23]$$

Thus, we partially proved the result. It remains to be shown that the following

$$\gamma \geq \frac{\alpha\delta(\delta(N-3)-1)}{N-3}$$

is a necessary condition. As shown in Example 1, the necessary conditions derived in [23] do not guarantee that V_i attains its maximum in $\mathbf{a}_i^\star$. In particular, consider $\Delta = V_i(x=0, y=N-2, \mathbf{a}_{-i}^\star) - V_i^\star$, then using [21] and [22] we obtain

$$\Delta = \alpha\left(1+\delta(N-2)+2\delta^2(N-2)\right) + \\ -\gamma(N-2) - \left(\alpha\left(1+\delta(N-1)+\delta^2(N-1)\right)-\gamma\right).$$

Imposing $\Delta \leq 0$ yields to a second lower bound for γ

$$\gamma \geq \alpha\delta\left(\delta - \frac{1}{N-3}\right), \qquad [24]$$

for otherwise, links are sufficiently cheap that a peripheral agent would eventually prefer to disconnect from the central agent and connect to all the other nodes. By doing so, she can increase the number of incoming paths of length 3, while simply losing a path of length 2. Moreover, note that [24] restricts the range of γ derived in [23] with the VI approach whenever $2\beta \leq -\alpha\delta/(N-3)$.

Therefore, thanks to [23] and [24] we finally proved that the necessary conditions are as follows:

$$\gamma \geq \max\left\{\alpha\delta^2+2\beta, \alpha\delta\left(\delta-\frac{1}{N-3}\right)\right\} = \underline{\gamma}_{NE} \\ \gamma \leq \alpha\delta(1+\delta) = \bar{\gamma}_{NE}. \qquad [25]$$

$(\Longleftarrow)$: We verify that the necessary conditions as derived in [25] are also sufficient. Firstly, consider the central node and recall the expression of the payoff function $V_1(\mathbf{a}_1, \mathbf{a}_{-1}^\star)$ as in [20]. One can notice that this expression is simply a function of $z = \sum_{k\neq 1} a_{1k} \in [0, N-1]$ and can be re-written as:

$$V_1(z, \mathbf{a}_{-1}^\star) = \alpha\left(N-1+\delta z+\delta^2(N-1)z\right) - \gamma z = \\ = \alpha(N-1) + \alpha\delta(1+\delta(N-1)-\gamma)z.$$

When $\gamma \leq \bar{\gamma}_{NE}$, this is a linear non-decreasing function of z, thus it achieves its maximum at $V_1^\star$, when $z = N-1$.

Conversely, for a peripheral node $i \neq 1$, we prove that $\mathbf{a}_i^\star$ is the best response for agent i when the necessary conditions [25] are satisfied, namely that

$$V_i^\star = \max_{\mathbf{a}_i \in \mathscr{A}} V_i(\mathbf{a}_i, \mathbf{a}_{-i}^\star).$$

Using the notation introduced previously in [21], we can rewrite the problem in the following form

$$V_i^\star = \max_{x\in[0,1], y\in[0,N-2]} V_i(x, y, \mathbf{a}_{-i}^\star).$$

This is a constrained maximization problem and the utility function is in general indefinite as the eigenvalues of its Hessian matrix

$$H = \begin{bmatrix} 0 & 2\beta \\ 2\beta & 0 \end{bmatrix}$$

are $\pm 2\beta$. Thus, the utility function cannot attain its maxima in the interior of the domain, but only at the boundaries, namely on some $(x^\star, y^\star)$ with $x^\star \in \{0,1\}$ or $y^\star \in \{0, N-2\}$. Moreover, it can be seen that the utility function restricted on the boundaries is a linear function, either in x or in y. Therefore the constrained maxima can be taken only on the corner points, i.e., in the set $\{(0,0), (0,N-2), (1,N-2), (1,0)\}$. Then, thanks to the VI necessary conditions derived in [23], we immediately verify that a strict maximum cannot be attained neither in $(1, N-2)$, as

$$V_i(x=1, y, \mathbf{a}_{-i}^\star) = \alpha(1+\delta(1+\delta)(N-1)) - \gamma + \\ + \underbrace{\left(\alpha\delta^2+2\beta-\gamma\right)}_{\leq 0} y$$

is a non-increasing function of y, nor in $(0,0)$ as

$$V_i(x, y=0, \mathbf{a}_{-i}^\star) = \alpha(1+\delta(1+\delta)(N-2)) + \\ + \underbrace{\left(\alpha\delta(1+\delta)-\gamma\right)}_{\geq 0} x$$

is a non-decreasing function of x.

Moreover, thanks to the additional necessary condition [24], we also have that such a strict maximum cannot be attained in $(0, N-2)$. This finally allows us to conclude that, if the set of necessary condition [25] is satisfied, then any peripheral node at the star network topology is already playing her best response strategy. Together with the previous result on the central node, this concludes the proof of the sufficiency of the conditions for the Nash equilibrium of the star network topology.

b) We now consider the pairwise-Nash equilibrium conditions of the star network.

$(\Longrightarrow)$: According to the result in Theorem 5 and recalling Remark 2, the necessary and sufficient condition for satisfying the NE property (C2) of the PNE-stability definition is

$$\alpha\delta^2 + 2\beta \leq \gamma \leq \alpha\delta(1+\delta) = \bar{\gamma}_{NE},$$

as derived in [23]. Similarly to the previous case (the balanced complete bipartite topology), we need to introduce an extra necessary condition in order to prevent cooperation between agents in the periphery bringing to the formation of favorable mutual ties. In order to formally derive this

condition, consider two distinct agents i, j different from the central one. At the equilibrium topology, these agents are not linked and their payoff as function of the meeting strategy (a_{ij}, a_{ji}) reads as

$$\begin{aligned} & V_i(a_{ij}, a_{ji}, \mathbf{a}^\star_{-(i,j)}) = \\ & = \alpha\left(1 + a_{ji} + \delta\left(N - 1 + (1 + a_{ij})\, a_{ji}\right)\right) + \\ & + \alpha\delta^2\left(N - 1 + a_{ij} + N a_{ji} + a_{ij}a_{ji} + a_{ij}a_{ji}^2\right) + \\ & + 2\beta a_{ij} - \gamma(1 + a_{ij}), \end{aligned} \qquad [26]$$

for agent i, and similarly for agent j. Example 2 suggests to consider as educated counterexample the mutual strategy $(1,1)$. Then the Pareto optimality condition (C3) requires that agents i and j cannot simultaneously be better off by playing this strategy. Given the symmetry of the topology and of the mutual strategy $(1,1)$, then $V_i\left(1, 1, \mathbf{a}^\star_{-(i,j)}\right) = V_j\left(1, 1, \mathbf{a}^\star_{-(i,j)}\right)$. Therefore, it is sufficient to consider agent i's payoff function. Computing

$$\begin{aligned} \Delta &= V_i\left(1, 1, \mathbf{a}^\star_{-(i,j)}\right) - V_i^\star = \\ &= \alpha\left(1 + 2\delta + (N+3)\,\delta^2\right) + 2\beta - \gamma \end{aligned}$$

and imposing $\Delta \leq 0$, leads precisely to the following necessary condition

$$\gamma \geq \alpha\left(1 + 2\delta + (N+3)\,\delta^2\right) + 2\beta = \underline{\gamma}_{PNE}. \qquad [27]$$

For otherwise, the cooperative strategy $(1,1)$ makes both agents better off. Finally, comparing the result just obtained with the lower bound in the necessary condition [23], we conclude that the necessary condition for satisfying the PNE for the star topology is precisely:

$$\alpha\left(1 + 2\delta + (N+3)\,\delta^2\right) + 2\beta \leq \gamma \leq \alpha\delta(1+\delta),$$

as in the statement of the theorem.

$(\Longleftarrow)$: We finally prove sufficiency of these conditions. We already know that the necessary condition is also a sufficient condition for the NE property (C2). It therefore remains to verify the Pareto optimality condition. Given the symmetry of the topology, it is sufficient to pick

- the pair $(1, j)$ with $j \neq 1$,
- the pair (i, j) with i and j distinct and different from 1.

However, for any pair $(1, j)$ of the first type, the equilibrium strategy is precisely $(a^\star_{1j}, a^\star_{j1}) = (1,1)$, therefore we can invoke Theorem 6 (i) and conclude that condition (C3) is satisfied for these pairs. Thus, it remains to consider a pair of agents in the periphery: let $i, j \neq 1$. The payoff function of agent i (j is analogous) as a function of the meeting strategy (a_{ij}, a_{ji}) was derived in [26], therefore we can compute

$$\begin{aligned} & \Delta(a_{ij}, a_{ji}) = \\ & V_i\left(a_{ij}, a_{ji}, \mathbf{a}^\star_{-(i,j)}\right) + V_j\left(a_{ij}, a_{ji}, \mathbf{a}^\star_{-(i,j)}\right) - \left(V_i^\star + V_j^\star\right) = \\ & = \alpha\left(\cancel{2} + a_{ji} + a_{ji} + \delta\left(\cancel{2(N-1)} + a_{ij} + a_{ji} + 2a_{ij}a_{ji}\right)\right) + \\ & + \alpha\delta^2\left(\cancel{2(N-1)} + (N+1)\,a_{ij} + (N+1)\,a_{ji}\right) + \\ & + \alpha\delta^2\left(2 + a_{ij} + a_{ji}\right) a_{ij}a_{ji} + \\ & + 2\beta\left(a_{ij} + a_{ji}\right) - \gamma\left(\cancel{2} + a_{ij} + a_{ji}\right) + \\ & \cancel{-2\alpha\left(1 + \delta(N-1) + \delta^2(N-1)\right) + 2\gamma} = \\ & = \alpha\left(a_{ji} + a_{ji} + \delta\left(a_{ij} + a_{ji} + 2a_{ij}a_{ji}\right)\right) + \\ & + \alpha\delta^2\left((N+1)\,a_{ij} + (N+1)\,a_{ji} + (2 + a_{ij} + a_{ji})\,a_{ij}a_{ji}\right) + \\ & + (2\beta - \gamma)\left(a_{ij} + a_{ji}\right). \end{aligned}$$

By using the necessary condition [27], namely

$$2\beta - \gamma \leq -\alpha\left(1 + 2\delta + (N+3)\,\delta^2\right)$$

$$\begin{aligned} & \Delta(a_{ij}, a_{ji})] \leq \\ & \leq \alpha\left(a_{ji} + a_{ji} + \delta\left(a_{ij} + a_{ji} + 2a_{ij}a_{ji}\right)\right) + \\ & + \alpha\delta^2\left((N+1)\,a_{ij} + (N+1)\,a_{ji} + (2 + a_{ij} + a_{ji})\,a_{ij}a_{ji}\right) + \\ & - \alpha\left(1 + 2\delta + (N+3)\,\delta^2\right)\left(a_{ij} + a_{ji}\right) = \\ & = \alpha\delta\underbrace{\left(2a_{ij}a_{ji} - (a_{ij} + a_{ji})\right)}_{\leq 0} + \\ & + \alpha\delta^2\left[(2 + a_{ij} + a_{ji})\,a_{ij}a_{ji} - 2\,(a_{ij} + a_{ji})\right] = \\ & \leq \alpha\delta^2\left[\underbrace{a_{ij}\,(a_{ji} - 1)}_{\leq 0} + \underbrace{a_{ji}\,(a_{ij} - 1)}_{\leq 0} + \underbrace{(a_{ij} + a_{ji})\,(a_{ij}a_{ji} - 1)}_{\leq 0}\right] \\ & \leq 0. \end{aligned}$$

Invoking Theorem 6 (ii), we show that the necessary conditions are also sufficient in order to guarantee the Pareto optimality condition for this pair, thus concluding the proof for the star network topology.

□

Example 1. Consider the following settings: $N = 50$ and $\delta = 0.5$. Then, $\alpha = 9$, $\beta = -1$, $\gamma = 1$ satisfy the necessary condition [23]. As shown in Supplementary Fig. 3, the maximum of the utility function for a peripheral agent i is taken when $y = N - 2$ and $x = 0$. Thus, $\mathbf{a}^\star_i$ does not satisfy the Nash equilibrium condition, as agent i is better off by removing the link towards the center of the star and simultaneously linking to all the other peripheral nodes.

This counterexample shows that we need to introduce further necessary conditions which can guarantee that the maximum of V_i is taken exactly at $V_i^\star = V_i(x = 1, y = 0, \mathbf{a}^\star_{-i})$, and it suggests us to consider as educated counterexample the value of $V_i(x = 0, y = N-2, \mathbf{a}^\star_{-i})$.

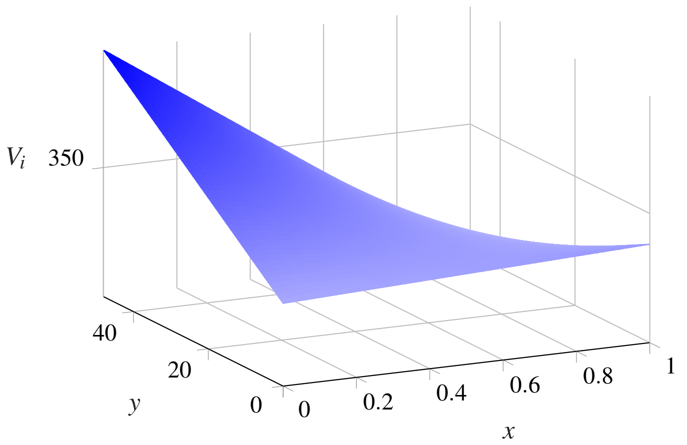

Supplementary Figure 3. Utility function of a peripheral node i when [23] is satisfied.

Example 2. In this example, we want to consider the case in which the selfish condition (C2) is satisfied, while the Pareto optimality condition (C3) is not. We consider a star network, with the following settings, $N = 50$ agents, $\alpha = 3$, $\beta = -10$, $\gamma = 1$, $\delta = 0.5$. Here, two disconnected agents i and j in the periphery have no selfish incentive in changing strategy. As shown in Supplementary Fig. 4, $a^\star_{ij} = a^\star_{ji} = 0$ guarantees the Nash property (C2). However, the Nash equilibrium strategy does not satisfy (C3), as it does not belong to the Pareto optimal front: with this settings, actors in the periphery find beneficial to become mutually connected. This shows that selfish and cooperative incentives are not always aligned.

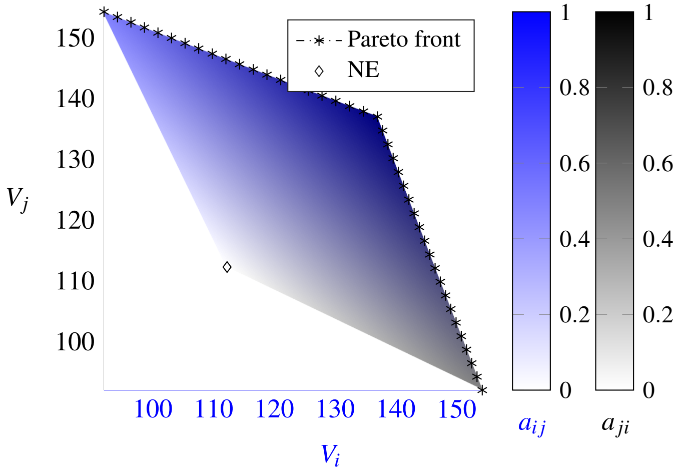

Supplementary Figure 4. Star Network of $N = 50$ agents, with $\alpha = 3$, $\beta = -10$, $\gamma = 1$ and $\delta = 0.5$. The utility functions of two peripheral nodes i and j are plotted. The corresponding point in the parameter space is such that the star network satisfies (C2) but not (C3). Indeed, agents cannot selfishly improve their payoff function, but they can both be better off by means of cooperation, i.e. connecting one another.

Supplementary Note 3. *Behavior Estimation Method*

In our behavior estimation method, we consider agents with heterogeneous individual preferences' sets $P_i = \{\alpha_i, \beta_i, \gamma_i, \delta_i\}$. We assume to observe a network $\mathscr{G}^\star$ of N agents, where the actions $\mathbf{a}^\star_i$ of the agents are "approximately" a Nash equilibrium with respect to the payoff functions $V_i(\mathbf{a}_i, \mathbf{a}_{-i}, P_i)$ which depends on some unknown parameters P_i. The term "approximately" derives from the fact that we introduce an error term to account for bounded rationality, which might justify a number of violations of the Nash equilibrium conditions. Our goal is to estimate the heterogeneous individual parameters from the observed state of the network. This learning and inference problem can be cast as an inverse optimization problem over candidate objective functions, which have a wide spectrum of applications spanning several disciplines from econometric and operations research to engineering and biology[3]. Inverse optimization methods are available for combinatorial optimization problems[4], and linear[5] and conic[6] programming. In our case, we start off by considering the difference in the payoff to player i when deviating to a different strategy $\mathbf{a}_i \in \mathscr{A}$,

$$e_i(\mathbf{a}_i, P_i) := \frac{1}{\gamma_i}\left(V_i\left(\mathbf{a}_i, \mathbf{a}^\star_{-i}, P_i\right) - V_i\left(\mathbf{a}^\star_i, \mathbf{a}^\star_{-i}, P_i\right)\right). \qquad [28]$$

which can be rewritten, using the alternative formulation of the payoff function, as a linear function in the parameter θ_i:

$$\begin{aligned} e_i(\mathbf{a}_i, \theta_i) = \theta_{i,1}&\left(rec\left(\mathbf{a}_i, \mathbf{a}^\star_{-i}\right) - rec\left(\mathbf{a}^\star_i, \mathbf{a}^\star_{-i}\right)\right) + \\ &+ \theta_{i,2}\left(rec\left(\mathbf{a}_i, \mathbf{a}^\star_{-i}\right) \cdot indeg\left(\mathbf{a}^\star_{-i}\right) + cycles\left(\mathbf{a}_i, \mathbf{a}^\star_{-i}\right) + \right.\\ &\left. - \left(rec\left(\mathbf{a}^\star_i, \mathbf{a}^\star_{-i}\right) \cdot indeg\left(\mathbf{a}^\star_{-i}\right) + cycles\left(\mathbf{a}^\star_i, \mathbf{a}^\star_{-i}\right)\right)\right) \\ &+ \theta_{i,3} \cdot \left(u_i(\mathbf{a}_i, \mathbf{a}^\star_{-i}) - u_i(\mathbf{a}^\star_i, \mathbf{a}^\star_{-i})\right) + \\ &- \left(c_i(\mathbf{a}_i) - c_i(\mathbf{a}_i^\star)\right). \end{aligned}$$

Furthermore, we can introduce the following notation:

$$\begin{aligned} x_{i,1}(\mathbf{a}_i) =& rec\left(\mathbf{a}_i, \mathbf{a}^\star_{-i}\right) - rec\left(\mathbf{a}^\star_i, \mathbf{a}^\star_{-i}\right), \\ x_{i,2}(\mathbf{a}_i) =& \left(rec\left(\mathbf{a}_i, \mathbf{a}^\star_{-i}\right) - rec\left(\mathbf{a}^\star_i, \mathbf{a}^\star_{-i}\right)\right) \cdot indeg\left(\mathbf{a}^\star_{-i}\right) + \\ &+ cycles\left(\mathbf{a}_i, \mathbf{a}^\star_{-i}\right) - cycles\left(\mathbf{a}^\star_i, \mathbf{a}^\star_{-i}\right), \\ x_{i,3}(\mathbf{a}_i) =& u_i(\mathbf{a}_i, \mathbf{a}^\star_{-i}) - u_i(\mathbf{a}^\star_i, \mathbf{a}^\star_{-i}), \\ y_i(\mathbf{a}_i) =& \left(c_i(\mathbf{a}_i) - c_i(\mathbf{a}_i^\star)\right), \end{aligned} \qquad [29]$$

and rewrite the function e_i in a compact linear form:

$$e_i(\mathbf{a}_i, \theta_i) = \mathbf{x}_i(\mathbf{a}_i)\theta_i - y_i(\mathbf{a}_i), \qquad [30]$$

where we stacked $x_{i,1}, \ldots, x_{i,3}$ in a row vector $\mathbf{x}_i$.

The Nash equilibrium condition requires that, for all agents i, there exists no action $\mathbf{a}_i$ in the action set $\mathscr{A}$ that allows to strictly improve the payoff function of agent i, while the others' actions $\mathbf{a}^\star_{-i}$ remain fixed. Conversely, the Nash equilibrium condition is violated for agent i if and only if there exists an action $\mathbf{a}_i \neq \mathbf{a}^\star_i$ such that

$$V_i\left(\mathbf{a}_i, \mathbf{a}^\star_{-i}, P_i\right) - V_i\left(\mathbf{a}_i{}^\star, \mathbf{a}^\star_{-i}, P_i\right) > 0.$$

Thus, the function $e_i(\mathbf{a}_i, \theta_i)$ takes positive values whenever the preference θ_i is such that the Nash equilibrium constraint is violated by the action $\mathbf{a}_i$. This positive violations can be regarded as errors due to, for instance, the bounded rationality of the agents. Hence we introduce an error term defined as:

$$e_i^+(\mathbf{a}_i, \theta_i) := \max\{0, e_i(\mathbf{a}_i, \theta_i)\}.$$

A natural measure of the distance from the Nash equilibrium condition can be obtained integrating the square of the error terms $e_i^+(\mathbf{a}_i, \theta_i)$ over the entire action set

$$d_i(\theta_i) := \left(\int_{\mathscr{A}} e_i^+(\mathbf{a}_i, \theta_i)^2 d\mathbf{a}_i\right)^{1/2}, \quad [31]$$

which corresponds to the $L_2(\mathscr{A})$ norm of the (positive) error function. In other words, $d_i(\theta_i)$ is the average Euclidean distance from NE. Note that alternative definitions could be used. For instance the $L_\infty(\mathscr{A})$ norm of the error function

$$\left|\left|e_i^+(\mathbf{a}_i, \theta_i)\right|\right|_{L_\infty(\mathscr{A})} := \max_{\mathbf{a}_i \in \mathscr{A}} \left|e_i^+(\mathbf{a}_i, \theta_i)\right|$$

has a nice interpretation in terms of ε-Nash equilibrium: for a fixed set of individual parameters $\left\{\hat{\theta}_1, \dots, \hat{\theta}_N\right\}$, let

$$\varepsilon = \max_{i=1,\dots,N} \left|\left|e_i^+(\mathbf{a}_i, \hat{\theta}_i)\right|\right|_{L_\infty(\mathscr{A})},$$

then the network $\mathscr{G}^\star$ is an ε-Nash equilibrium, i.e., for all agents i,

$$V_i\left(\mathbf{a}_i, \mathbf{a}_{-i}^\star, \hat{\theta}_i\right) \le V_i\left(\mathbf{a}_i^\star, \mathbf{a}_{-i}^\star, \hat{\theta}_i\right) + \varepsilon, \ \forall \mathbf{a}_i \in \mathscr{A}. \quad [32]$$

On the other hand, the L_2 norm has the following two desirable properties: it can be shown that it behaves well with respect to θ_i, and it induces a nice interpretation in terms of statistical regression. In order to make the first property more formal, we start by proving the following Theorem.

Theorem 11. *Let $f(\mathbf{x}, \theta) : \mathbb{R}^n \times \mathbb{R}^p \to \mathbb{R}$ be a continuous function of $\mathbf{x} \in \mathbb{R}^n$ and $\theta \in \mathbb{R}^p$. Moreover, assume f to be linear in θ, and let $\mathscr{X}$ be a compact subset of $\mathbb{R}^n$. Consider the following function:*

$$F(\theta) := \int_{\mathscr{X}} (\max\{0, f(\mathbf{x}, \theta)\})^2 d\mathbf{x}.$$

Then F is continuously differentiable, and its gradient is

$$\nabla_\theta F(\theta) = \int_{\mathscr{X}} 2\nabla_\theta (f(\mathbf{x}, \theta)) \max\{0, f(\mathbf{x}, \theta)\} d\mathbf{x}. \quad [33]$$

Moreover, F is a convex function.

Proof. Firstly, note that $(\max\{0, f(\mathbf{x}, \theta)\})^2$ is a function of class C^1 with respect to θ, for all $\mathbf{x} \in \mathbb{R}^n$. Namely, its gradient given by

$$\nabla_\theta\left((\max\{0, f(\mathbf{x}, \theta)\})^2\right) = \begin{cases} 0, & \text{if } f(\mathbf{x}, \theta) < 0; \\ f(\mathbf{x}, \theta)\nabla_\theta f(\mathbf{x}, \theta), & \text{otherwise,} \end{cases}$$

is a continuous function in θ, since (i)$f(\mathbf{x}, \theta)$ is linear in θ and thus $\nabla_\theta f(\mathbf{x}, \theta)$ is continuous in θ; and (ii) continuity is also preserved where $f(\mathbf{x}, \theta) = 0$. Then, we compute partial derivatives of F with respect to θ_i, with $i = 1, \dots, p$.

$$\frac{\partial F(\theta)}{\partial \theta_i} = \frac{\partial}{\partial \theta_i} \int_{\mathscr{X}} (\max\{0, f(\mathbf{x}, \theta)\})^2 d\mathbf{x}.$$

Since the integrand function is of class C^1 with respect to θ, we can apply Leibniz integral rule:

$$\frac{\partial F(\theta)}{\partial \theta_i} = \int_{\mathscr{X}} \frac{\partial}{\partial \theta_i} (\max\{0, f(\mathbf{x}, \theta)\})^2 d\mathbf{x}.$$

Consider $\mathscr{X}_+(\theta) = \{\mathbf{x} \in \mathscr{X} | f(\mathbf{x}, \theta) > 0\}$ and $\mathscr{X}_-(\theta) = \{\mathbf{x} \in \mathscr{X} | f(\mathbf{x}, \theta) \le 0\}$, then

$$\begin{aligned} \frac{\partial F(\theta)}{\partial \theta_i} &= \int_{\mathscr{X}_+(\theta)} \frac{\partial}{\partial \theta_i} (\max\{0, f(\mathbf{x}, \theta)\})^2 d\mathbf{x} + \\ &+ \int_{\mathscr{X}_-(\theta)} \frac{\partial}{\partial \theta_i} (\max\{0, f(\mathbf{x}, \theta)\})^2 d\mathbf{x} = \\ &= \int_{\mathscr{X}_+(\theta)} \frac{\partial}{\partial \theta_i} (f(\mathbf{x}, \theta))^2 d\mathbf{x} + \int_{\mathscr{X}_-(\theta)} \frac{\partial}{\partial \theta_i} 0 d\mathbf{x} = \\ &= \int_{\mathscr{X}_+(\theta)} 2f(\mathbf{x}, \theta) \frac{\partial}{\partial \theta_i} (f(\mathbf{x}, \theta)) d\mathbf{x} = \\ &= \int_{\mathscr{X}} 2\max\{0, f(\mathbf{x}, \theta)\} \frac{\partial}{\partial \theta_i} (f(\mathbf{x}, \theta)) d\mathbf{x}. \end{aligned}$$

Note that $\frac{\partial}{\partial \theta_i}(f(\theta))$ is independent of θ as $f(\mathbf{x}, \theta)$ is linear in θ. Thus, $\frac{\partial F(\theta)}{\partial \theta_i}$ is continuous. In vector form, the gradient reads as follows

$$\nabla_\theta F(\theta) = \int_{\mathscr{X}} 2\nabla_\theta (f(\mathbf{x}, \theta)) \max\{0, f(\mathbf{x}, \theta)\} d\mathbf{x}.$$

This concludes the first part of the proof.

In the next step, we aim at computing the Hessian matrix of F, namely

$$\frac{\partial^2 F(\theta)}{\partial \theta_j \partial \theta_i} = \frac{\partial}{\partial \theta_j} \left(\int_{\mathscr{X}} 2\max\{0, f(\mathbf{x}, \theta)\} \frac{\partial}{\partial \theta_i} (f(\mathbf{x}, \theta)) d\mathbf{x}\right).$$

In order to be able to apply again the Leibniz rule, we need the integrand function to be of class C^1 with respect to θ. Let $h_i(\mathbf{x}) := \max\{0, f(\mathbf{x}, \theta)\} \frac{\partial f(\mathbf{x}, \theta)}{\partial \theta_i}$, for $i = 1, \dots, p$. Unfortunately,

$$\frac{\partial h_i(\mathbf{x}, \theta)}{\partial \theta_j} = \begin{cases} 0 & \text{if } f(\mathbf{x}, \theta) < 0, \\ \frac{\partial f(\mathbf{x}, \theta)}{\partial \theta_i} \frac{\partial f(\mathbf{x}, \theta)}{\partial \theta_j} & \text{otherwise,} \end{cases}$$

is only piecewise-continuous, with discontinuity set $\mathscr{X}_0(\theta) = \{\mathbf{x} \in \mathscr{X}, f(\mathbf{x}, \theta) = 0\}$. Note, though, that for all θ_j, $\frac{\partial h_i(\mathbf{x}, \theta)}{\partial \theta_j}$ exists for all $\mathbf{x} \in \mathscr{X} \setminus \mathscr{X}_0(\theta)$, and there exists an integrable function $p_i : \mathscr{X} \to \mathbb{R}$ such that $\left|\frac{\partial h_i(\mathbf{x}, \theta)}{\partial \theta_j}\right| \le p_i(\mathbf{x})$ for all $\theta \in \Theta$, since the partial derivatives of f with respect to all the components of θ are linear functions of $\mathbf{x}$ and independent of

θ. Thanks to these observations, the integrand function admits bounded distributional derivative, thus we can apply the dominated convergence theorem (see, for instance,[7]) which yields

$$\begin{aligned}\frac{\partial^2 F(\theta)}{\partial\theta_j\partial\theta_i} &= \frac{\partial}{\partial\theta_j}\left(\int_{\mathscr{X}} 2\max\{0, f(\mathbf{x},\theta)\}\frac{\partial}{\partial\theta_i}(f(\mathbf{x},\theta))\,d\mathbf{x}\right) =\\ &= 2\int_{\mathscr{X}}\frac{\partial}{\partial\theta_j}\left(\max\{0,f(\mathbf{x},\theta)\}\frac{\partial}{\partial\theta_i}(f(\mathbf{x},\theta))\right)d\mathbf{x} =\\ &= 2\int_{\mathscr{X}_+(\theta)}\frac{\partial}{\partial\theta_j}\left(\max\{0,f(\mathbf{x},\theta)\}\frac{\partial}{\partial\theta_i}(f(\mathbf{x},\theta))\right)d\mathbf{x}+\\ &+2\int_{\mathscr{X}_-(\theta)}\frac{\partial}{\partial\theta_j}\left(\max\{0,f(\mathbf{x},\theta)\}\frac{\partial}{\partial\theta_i}(f(\mathbf{x},\theta))\right)d\mathbf{x} =\\ &= 2\int_{\mathscr{X}_+(\theta)}\frac{\partial}{\partial\theta_j}\left(f(\mathbf{x},\theta)\frac{\partial}{\partial\theta_i}(f(\mathbf{x},\theta))\right)d\mathbf{x} =\\ &= 2\int_{\mathscr{X}_+(\theta)}\frac{\partial f(\mathbf{x},\theta)}{\partial\theta_j}\frac{\partial f(\mathbf{x},\theta)}{\partial\theta_i}+f(\mathbf{x},\theta)\frac{\partial^2 f(\mathbf{x},\theta)}{\partial\theta_i\partial\theta_j}d\mathbf{x} =\\ &= 2\int_{\mathscr{X}_+(\theta)}\frac{\partial f(\mathbf{x},\theta)}{\partial\theta_j}\frac{\partial f(\mathbf{x},\theta)}{\partial\theta_i}d\mathbf{x},\end{aligned}$$

where the last step is due to the fact that $f(\mathbf{x},\theta)$ is linear in θ. Thus, the Hessian matrix of F, which reads as

$$\mathbf{H}(F(\theta)) = 2\int_{\mathscr{X}_+(\theta)}\nabla_\theta(f(\mathbf{x},\theta))\nabla_\theta(f(\mathbf{x},\theta))^T\,d\mathbf{x},$$

is positive semi-definite, as for any $\mathbf{y}\in R^p$

$$\begin{aligned}\mathbf{y}^T\mathbf{H}(F(\theta))\mathbf{y} &= \mathbf{y}^T 2\int_{\mathscr{X}_+(\theta)}\nabla_\theta(f(\mathbf{x},\theta))\nabla_\theta(f(\mathbf{x},\theta))^T\,d\mathbf{x}\mathbf{y} =\\ &= 2\int_{\mathscr{X}_+(\theta)}\mathbf{y}^T\nabla_\theta(f(\mathbf{x},\theta))\nabla_\theta(f(\mathbf{x},\theta))^T\,\mathbf{y}d\mathbf{x} =\\ &= 2\int_{\mathscr{X}_+(\theta)}\left|\left|\mathbf{y}^T\nabla_\theta(f(\mathbf{x},\theta))\right|\right|^2 d\mathbf{x}\geq 0.\end{aligned}$$

Thus, F is convex. □

The result of the theorem can then be directly applied to the distance function $d_i(\theta_i)$ defined in [31]. This guarantees that $d_i(\theta_i)^2$ is a convex function and therefore the following problem is well posed.

Problem (Minimum NE-Distance Problem)**.** Given a network $\mathscr{G}^\star$ of N agents, for all agents i find the vectors of preferences $\theta_i^\star$ such that

$$\theta_i^\star \in \arg\min_{\theta_i\in\Theta} d_i^2(\theta_i),$$

where d_i is the distance function previously defined, namely

$$d_i(\theta_i) := \left(\int_{\mathscr{A}} e_i^+(\mathbf{a}_i,\theta_i)^2 d\mathbf{a}_i\right)^{1/2}.$$

Note that d_i is a non-negative function, thus the minimizers of d_i^2 correspond to the minimizers of d_i. In order to approach the problem, we define the set $\Theta_{i,0}\subseteq\Theta$ as

$$\Theta_{i,0} := \{\theta_i\in\Theta, \text{ s.t. } \forall\mathbf{a}_i\in\mathscr{A}, e_i(\mathbf{a}_i,\theta_i)\leq 0\}. \qquad [34]$$

Then we can distinguish two cases:

1. $\Theta_{i,0}$ is non-empty. Then by [34], for all θ_i in $\Theta_{i,0}$, and for all $\mathbf{a}_i\in\mathscr{A}$, it holds $e_i(\mathbf{a}_i,\theta_i)\leq 0$. By definition of the error function in [28], for all $\theta_i\in\Theta_{i,0}$ this implies

   $$V_i\left(\mathbf{a}_i,\mathbf{a}^\star_{-i},\theta_i\right)\leq V_i\left(\mathbf{a}_i{}^\star,\mathbf{a}^\star_{-i},\theta_i\right),\ \forall\mathbf{a}_i\in\mathscr{A},$$

   i.e., the Nash equilibrium conditions (of agent i) are satisfied exactly. Note that, For these θ_i, $e_i^+(\mathbf{a}_i,\theta_i)=0$ for all $\mathbf{a}_i\in\mathscr{A}$, thus the minimum NE-distance is 0.

2. $\Theta_{i,0}$ is empty. In this case, there exists no θ_i that can satisfy all the Nash equilibrium conditions exactly. Yet, all $\theta_i\in\Theta$ satisfy the following condition: $\forall\mathbf{a}_i\in\mathscr{A}$,

   $$V_i\left(\mathbf{a}_i,\mathbf{a}^\star_{-i},\theta_i\right)\leq V_i\left(\mathbf{a}_i{}^\star,\mathbf{a}^\star_{-i},\theta_i\right)+e_i^+\left(\mathbf{a}_i,\mathbf{a}^\star_{-i},\theta_i\right),$$

   which, in fact, is an equality condition. In other words, the minimum NE-Distance problem looks for the θ_i that requires the minimum deviation with respect to the Nash equilibrium condition, in terms of the L_2 norm[1].

Perfect Nash equilibrium

We start by discussing the first case, i.e., the "perfect Nash equilibrium". In this case, solving the Minimum-NE Distance problem reduces to finding the non-empty region $\Theta_{i,0}$. This region is a convex subset of Θ. Let $\theta_{i,1}$ and $\theta_{i,2}\in\Theta_{i,0}$, and consider a convex combination $\tilde{\theta}_i = t\theta_{i,1}+(1-t)\theta_{i,2}$, where $t\in[0,1]$. Then, by linearity of $e_i(\mathbf{a}_i,\theta_i)$ with respect to θ_i (see [30]),

$$e_i(\mathbf{a}_i,\tilde{\theta}_i) = te_i(\mathbf{a}_i,\theta_{i,1})+(1-t)e_i(\mathbf{a}_i,\theta_{i,2})\leq 0, \forall\mathbf{a}_i\in\mathscr{A},$$

i.e., $\tilde{\theta}_i\in\Theta_{i,0}$, as it satisfies the definition in [34]. Moreover, due to the linearity with respect to θ (see [30]), the region $\Theta_{i,0}$ can be rewritten as

$$\Theta_{i,0} := \{\theta_i\in\Theta, \text{ s.t. } \forall\mathbf{a}_i\in\mathscr{A}, \mathbf{x}_i(\mathbf{a}_i)\theta_i - y_i(\mathbf{a}_i)\leq 0\},$$

thus it is the intersection of infinitely many half-planes. In the following, we provide a more tractable (finite) description of this region. Equivalently, we can write

$$\Theta_{i,0} := \left\{\theta_i\in\Theta, \text{ s.t. } \max_{\mathbf{a}_i\in\mathscr{A}}\left(\mathbf{x}_i(\mathbf{a}_i)\theta_i - y_i(\mathbf{a}_i)\right)\leq 0\right\}.$$

Note that the function $\mathbf{x}_i(\mathbf{a}_i)\theta_i - y_i(\mathbf{a}_i)$ is a combination of linear and mixed quadratic terms in the components of $\mathbf{a}_i$, but there are no pure quadratic terms of the form a_{ij}^2 (see [29] and the definitions in the article). Hence, we are dealing with a constrained maximization problem where the objective function is in general indefinite. In fact, the trace of the Hessian matrix is 0, thus the sum of the eigenvalues is 0 which implies that, if there exists a positive eigenvalues, there exists also a negative one. This implies that any critical point $\mathbf{a}_i$ in the compact set $\mathscr{A}=[0,1]^{N-1}$ is a saddle point, thus it cannot be a strict maximum. Since the

[1]In other words, it is similar to the ε-Nash equilibrium (see [32]) which requires minimum deviation in terms of the L_∞ norm. We emphasize that taking the L_2 norm allows to deal with a smooth function distance function, conversely when considering the L_∞ norm convexity does not hold in general.

function is continuous in a compact set, it must attain a maximum (and a minimum, Weierstrass theorem), and since it cannot be in the interior, it must be attained on the boundary. The boundary is composed of $2(N-1)$ sides, and each side is the compact set $[0,1]^{N-2}$. Once we restrict to the side, the Hessian matrix has the same properties. Thus, the maximum must be attained at the boundary of the side, which again is composed of $2(N-2)$ compact sets of type $[0,1]^{N-3}$. Proceeding by induction on N we reach the set of vertices, and we finally conclude that the maximum is attained at one of the vertices of the domain $\mathscr{A}$. Namely it suffices to consider

$$\Theta_{i,0} := \left\{ \theta_i \in \Theta, \text{ s.t. } \forall \mathbf{a}_i \in \mathscr{A}_{\{0,1\}}, \mathbf{x}_i(\mathbf{a}_i)\theta_i - y_i(\mathbf{a}_i) \leq 0 \right\},$$

where $\mathscr{A}_{\{0,1\}} = \{0,1\}^{N-1}$. Then, the set of inequalities is finite, though exponential in number, 2^{N-1}. It can be further reduced using, for instance, combinatorial uncrossing techniques (see[8]). Nonetheless, $\Theta_{i,0}$ is a (convex) polyhedron, which can be described in terms of vertices and rays.

Non-perfect Nash equilibrium

We then consider the second case, i.e. when $\Theta_{i,0}$ is empty. In this case, solving exactly the Minimum NE-Distance problem is not an easy task. Even though we showed that the objective function is convex, it is not strictly convex and the set of minimizers might not be a singleton, in the general case. This is due to the fact that the "max" operator induces some flat regions in the distance function. Moreover, the first order optimality conditions, which can be deduced from the gradient [33] derived in Theorem 11, namely

$$\nabla_{\theta_i}\left(d_i^2(\theta_i)\right) = 2\int_{\mathscr{A}} \nabla_{\theta_i}\left(e_i(\mathbf{a}_i,\theta_i)\right) \max\left\{0, e_i(\mathbf{a}_i,\theta_i)\right\} d\mathbf{a}_i,$$

are difficult to deal with because of the "max" function within the $(N-1)-$dimensional integral. The problem, though, becomes more tractable after discretization of the action space. For each agent i, consider a finite set of possible actions (samples) $\left\{\mathbf{a}_i^j\right\}_{j=1}^{n_i} \subset \mathscr{A}$, and let $e_i^j(\theta_i) = e_i(\mathbf{a}_i^j,\theta_i)$ and $e_i^{j,+}(\theta_i) = e_i^+(\mathbf{a}_i^j,\theta_i)$ be the corresponding error and positive error value at the samples. In practice, we usually consider

$$\mathbf{a}_i^j \in \mathscr{A}_{\{0,1\}} = \{0,1\}^{N-1} \qquad [35]$$

or a more refined regular mesh and we approximate the Riemann integral arbitrarily well by refining the mesh. Then we approximate the distance function as follows

$$\tilde{d}_i(\theta_i) := \left(\sum_{j=1}^{n_i}\left(e_i^{j,+}(\theta_i)\right)^2\right)^{1/2},$$

and we consider an approximated Minimum NE-Distance problem:

Problem (Discrete Minimum NE-Distance Problem)**.** Given a network $\mathscr{G}^\star$ of N agents, for all agents i find the vectors of preferences $\hat{\theta}_i$ such that

$$\hat{\theta}_i \in \arg\min_{\theta_i\in\Theta} \sum_{j=1}^{n_i}\left(e_i^{j,+}(\theta_i)\right)^2,$$

where

$$e_i^{j,+}(\mathbf{a}_i^j,\theta_i) = \max\left\{0, \mathbf{x}_i(\mathbf{a}_i^j)\theta_i - y_i(\mathbf{a}_i^j)\right\}, \ \forall j = 1,\ldots,n,$$

using the linear formulation of the residual function.

Such a "discretized" optimization problem inherits the same properties of the original problem, i.e., the convexity and smoothness (see Theorem 11) of the objective function, but also the possible non-uniqueness of the solution. Moreover, the gradient of the objective function can be found in closed form, and it reads as:

$$\nabla_{\theta_i}\left(\tilde{d}_i^{\,2}(\theta_i)\right) = 2\sum_{j=1}^{n_i} \nabla_{\theta_i} e_i^j(\theta_i) e_i^{j,+}(\theta_i).$$

Now, let $\mathbf{e}_i$, $\mathbf{e_i^+}$ and $\mathbf{y}_i$ be the stacked column vectors of respectively e_i^j, $e_i^{j,+}$ and $y_i(\mathbf{a}_i^j)$, and let $\mathbf{X}_i$ be the matrix of $\mathbf{x}_i(\mathbf{a}_i^j)$ stacked in rows.

Assume also, for now, that there exists an unconstrained minimum (note that in general $\theta_i \in \Theta = \mathbb{R}_+ \times \mathbb{R}_+ \times \mathbb{R}$). Then, the first-order optimality condition $\nabla_{\theta_i}\left(\tilde{d}_i^{\,2}(\theta_i)\right) = 0$ can be written as follows:

$$\mathbf{X}_i^T\left(max\left\{\mathbf{0}_{n_i\times 1}, \mathbf{X}_i\theta_i - \mathbf{y}_i\right\}\right) = 0. \qquad [36]$$

In the particular case where $\mathbf{X}_i\theta_i - \mathbf{y}_i = \mathbf{e}_i \geq \mathbf{0}_{n_i\times 1}$ componentwise, i.e., when $\mathbf{e}_i = \mathbf{e}_i^+$, we can neglect the "max" operator and the solution of the discrete Minimum NE-Distance problem is

$$\hat{\theta}_i = \left(\mathbf{X}_i^T\mathbf{X}_i\right)^{-1}\mathbf{X}_i^T\mathbf{y}_i.$$

However, this requires that $e_i^j \geq 0$, for all j. In other words, no action in the sample set strictly satisfies the Nash equilibrium condition (they all violate them).
In the more general case, though, there will be a subset of samples $J_i(\theta_i) \subset \{1,\ldots,n_i\}$ such that

$$\mathbf{x}_i^j\theta_i - y_i^j = e_i^j = e_i^{j,+} \geq 0 \quad \forall j \in J_i(\theta_i), \qquad [37]$$

while the remaining samples strictly satisfy the NE conditions, i.e., $e_i^j < 0$. We emphasize that, if the sample set contains all the vertices of $\mathscr{A}$ (for instance, the regular mesh defined in [35]), then $|J_i(\theta_i)| > 0$. For otherwise, we would fall in the previous case, the "Perfect Nash equilibrium".
Then, we define $\mathbf{X}_i(\theta_i)$ as the reduced matrix $\mathbf{X}_i$ where the rows corresponding to the indexes that do not belong to $J_i(\theta_i)$ have been removed. Similarly, we remove the corresponding rows in $\mathbf{e}_i$, $\mathbf{e}_i^+$ and $\mathbf{y}_i$. Then, we come back to the particular case where $\mathbf{X}_i(\theta_i)\theta_i - \mathbf{y}_i(\theta_i) = \mathbf{e}_i(\theta_i) \geq 0_{|J_i|\times 1}$, i.e., when $\mathbf{e}_i(\theta_i) = \mathbf{e}_i^+(\theta_i)$. At this point we can neglect the "max" operator and the solution of the discrete Minimum NE-Distance problem must still satisfy the first-order optimality conditions, thus it can be rewritten as:

$$\hat{\theta}_i = \left(\mathbf{X}_i(\hat{\theta}_i)^T\mathbf{X}_i(\hat{\theta}_i)\right)^{-1}\mathbf{X}_i(\hat{\theta}_i)^T\mathbf{y}_i(\hat{\theta}_i). \qquad [38]$$

Thanks to the convexity of the objective function, it is possible to find a solution $\hat{\theta}_i$ to the discrete Minimum NE-Distance problem, e.g., through gradient method.

Coming back to our assumption, more generally, the solution can be constrained to the boundaries of the search space. In general, the first order optimality conditions for minimizing a continuously differentiable function $F(\theta)$ require that:

$$\nabla_\theta F(\theta)^T\big|_{\hat{\theta}}(\mathbf{y}-\hat{\theta}) \geq 0, \qquad \forall \mathbf{y} \in \Theta, \tag{39}$$

which reduces to

$$\nabla_\theta F(\theta)\big|_{\hat{\theta}} = \mathbf{0},$$

in the unconstrained case. Even though from a computational perspective the projected gradient method still allows to find a solution $\hat{\theta}_i$ to our problem, we emphasize that the particular (closed-form) solution [38] can only be attained along the unconstrained directions.

Confidence intervals

In this section we would like to describe how we can derive confidence intervals on the solutions $\hat{\theta}_i$ by recasting the Discrete Minimum NE-Distance problem into an Ordinary Least Square (OLS) regression problem. In OLS, the linear model

$$\mathbf{y} = \mathbf{X}\theta - \varepsilon,$$

aims at explaining the observation ($\mathbf{y}$) through the dependency under a systematic component ($\mathbf{X}\theta$, where θ is a vector of parameters to be estimated, and $\mathbf{X}$ is the regression matrix made of n samples), and a stochastic component (ε). This is assumed to be an accurate reflection of the real world, and the goal of the least square regression problem is to obtain an estimate, generally indicated as $\hat{\theta}$, of the population parameters in the vector θ, which minimizes the sum of squared residuals $||\mathbf{e}||_2^2$, where the vector of residuals $\mathbf{e}$ is given by

$$\mathbf{e} = \mathbf{X}\theta - \mathbf{y}.$$

Therefore, the OLS problem can be formulated as follows:

Problem (Ordinary Least Square Problem)**.** Given a set of n samples $\{x_i\}_{i=1}^n$ and n observations $\{y_i\}_{i=1}^n$, where each scalar y_i is the response to the row vector x_i of values of p predictors (regressors) x_{ij} for $j = 1,\ldots,p$, find $\hat{\theta} \in \mathbb{R}^p$ such that

$$\hat{\theta} = \arg\min_\theta ||\mathbf{e}(\theta)||_2^2,$$

where $\mathbf{e} = \mathbf{X}\theta - \mathbf{y}$ is the vector of residuals.

Thanks to the convexity of the objective function, the analytical solution of the OLS problem can in general be found in closed form through the first order optimality condition, which yields

$$\hat{\theta} = (\mathbf{X}^T\mathbf{X})^{-1}\mathbf{X}^T\mathbf{y}. \tag{40}$$

Note that $\hat{\theta}$ is a linear estimator of θ with respect to the disturbance ε. Indeed, we can replace $\mathbf{y} = \mathbf{X}\theta - \varepsilon$ and obtain from the previous equation:

$$\hat{\theta} = (\mathbf{X}^T\mathbf{X})^{-1}\mathbf{X}^T(\mathbf{X}\theta - \varepsilon) = \theta - (\mathbf{X}^T\mathbf{X})^{-1}\mathbf{X}^T\varepsilon. \tag{41}$$

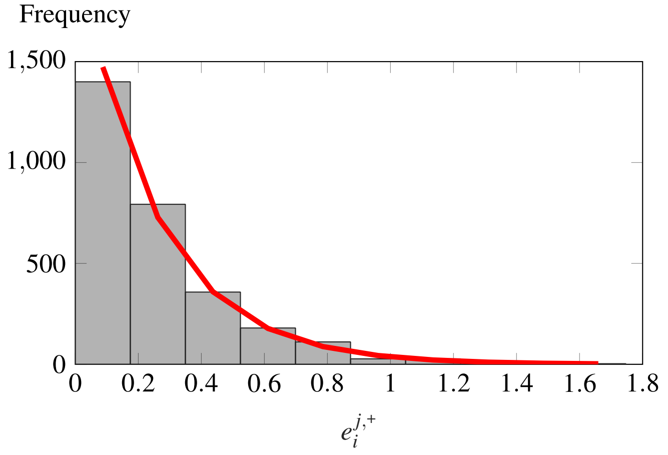

Supplementary Figure 5. Example of histogram of the errors $e_i^{j,+}$ used to estimate λ_i in the Australian bank network. Assuming the error are i.i.d. with exponential distribution of parameter λ_i, we use the realizations (errors) to compute the estimate $\hat{\lambda}_i = \frac{|J_i|}{\mathbf{1}^T\mathbf{e}_i^+}$. In the example shown, $|J_i| = 2912$ and $\hat{\lambda} = 4.036$. In red, the expected histogram of $|J_i|$ random realizations drawn independently from an exponential distribution of parameter $\hat{\lambda}_i$ shows a good match.

Moreover, if the disturbances are i.i.d. with 0 mean, then the OLS estimate is known to be unbiased[9], i.e. $E(\hat{\theta}) = \theta$.

Coming back to our problem, the similarity is evident both in the problem definition and in the solution [40][2], except for two observations: (i) the regression matrix $\mathbf{X}$ in our case depends on the solution; and (ii) the errors we observe are always non negative, thus we cannot assume they have zero mean. Concerning the first point, the only difference is that the solution needs to be found through (projected) gradient method rather than analytically. Nonetheless, once we redefine $\mathbf{X}_i$ as $\mathbf{X}_i(\hat{\theta}_i)$, the solution solves exactly the same first-order equation.

According to the second point, our estimate $\hat{\theta}_i$ is not unbiased. In order to correct it, we assume that the errors ε_i^j are i.i.d., following an exponential distribution of parameter λ_i, with $E\left[\varepsilon_i^j\right] = 1/\lambda_i$ and $var(\varepsilon_i^j) = 1/\lambda_i{}^2$. A concrete example (from the Australian bank test case) of the error histogram is shown in Supplementary Fig. 5.

Recalling [41], we can compute the bias of the OLS estimator $\hat{\theta}_i$

$$\begin{aligned} E\left[\hat{\theta}_i\right] - E\left[\theta_i\right] &= E\left[(\mathbf{X}_i^T\mathbf{X}_i)^{-1}\mathbf{X}_i^T\varepsilon_i\right] \\ &= (\mathbf{X}_i^T\mathbf{X}_i)^{-1}\mathbf{X}_i^T E\left[\varepsilon_i\right] = -\lambda_i{}^{-1}\mathbf{C}_i, \end{aligned}$$

where the $\mathbf{C}_i = (\mathbf{X}_i^T\mathbf{X}_i)^{-1}\mathbf{X}_i^T$ is the linearity matrix of the estimation dependency. Thus, the corrected (unbiased) estimator of θ_i is:

$$\begin{aligned} \tilde{\theta}_i &= \hat{\theta}_i + \lambda_i{}^{-1}\mathbf{C}_i \\ &= \theta_i + \lambda_i{}^{-1}\mathbf{C}_i - \mathbf{C}_i\varepsilon_i \\ &= E[\hat{\theta}_i] + 2\lambda_i{}^{-1}\mathbf{C}_i - \mathbf{C}_i\varepsilon_i. \end{aligned}$$

[2] More precisely, the analogy in the solution is valid for the unconstrained variables.

In order to be consistent with the usual notation, in the remaining part, as well as in the main paper, we keep using $\hat{\theta}_i$ to refer to the corrected unbiased estimator.

Finally, note that the unbiased estimator (renamed $\hat{\theta}_i$) is a linear combination of exponentially distributed variables. Although it might be challenging to compute the distribution in closed form, it is possible to simulate the distribution in order to find confidence intervals for the parameters. In particular, for a standard $100(1-\alpha)\%$ confidence intervals, it is sufficient to empirically compute the $\alpha/2$ and $1-\alpha/2$ quantiles of the distribution by taking a sufficiently large number of samples from the error distribution function. Concretely, we define a (large) number of samples M and a matrix $\mathbf{E} \in \mathbb{R}^{p\times M}$ such that each element is drawn from $\sim \mathrm{Exp}(\hat{\lambda}_i)$, where $\hat{\lambda}_i = \frac{|J_i|}{\mathbf{1}^T \mathbf{e}_\mathbf{i}^+}$ is an estimate of λ_i. From this, we can collect M samples of the distribution of $\hat{\theta}_i$ derived previously. Finally, we can define the empirical quantile function of the distribution of $\hat{\theta}_i$, and compute the $\alpha/2$ and $1-\alpha/2$ corresponding values to obtain the confidence intervals.

Supplementary Note 4. *Complex Networks Results*

The aim of this section is to provide additional information on the results presented in the section named "Inference of Behaviour for Complex Networks" in the main paper. Firstly, we report an extended description of the results of the Australian Bank network, then we focus on the application of the behavior estimation method to the Preferential Attachment model.

Australian Bank network

	Vertices			Rays		
	θ_1	θ_2	θ_3	θ_1	θ_2	θ_3
Deputy-M.	1	0	0	1	0	−1
				0	0	−1
Serv.-Ad.-1	0.5	0	0.33	0	0	−1
	0	0.11	0.30			
	0.8	0	0.2			
	0	0.14	0.14			
	1	0	0			
	0	0.12	0.27			
	0.11	0.11	0.26			
	0	0	0.44			
	0	0.05	0.38			
	0.2	0	0.4			
Serv.-Ad.-2	1	0	0	0	0	−1

Supplementary Table 1. Australian bank, polyhedra description of the region $\Theta_{i,0}$ of Deputy Manager and Service Advisers 1 and 2. For these 3 agents, there exists a non-empty convex subset of the parameter space whose elements (individual preferences) are compatible with the NE conditions.

Our second example of real-world network concerns the network of confiding relationships within an Australian bank, whose data have been collected by Pattison et al.[10]. In Fig. 8 in the article we present an overview of the result of the behavior estimation method applied to this example. For completeness, here we report the full results of the analysis. The behavior of three out of eleven agents, i.e., the Deputy Manager and Service Advisers 1 and 2, can be described exactly by the polyhedra $\Theta_{i,0}$ (see [34] and Table 1 for the description of $\Theta_{i,0}$ in terms of vertices and rays).

The remaining agents show contradictory actions which are incompatible with the Nash equilibrium conditions for any feasible values of the parameters. Thus, the analysis can only be performed accounting for an error term, modelling bounded rationality or noisy observations. In this case, we use the behavior estimation method to find a minimum NE-distance estimate and to build confidence intervals, as described in the previous section. Results are reported in Table 2. Note that, being the solution of the Minimum NE-distance problem not necessarily unique, we proceed as follows: we compute different solutions via projected gradient method (see previous section) starting from 8 different initial points, then, among these solutions, we select the one with minimum Euclidean norm, in order to ease the comparison. Note that, the same approach is used for the Medici test case.

Random Networks

To conclude, we would like to further elaborate on the application of the behavior estimation method to the Preferential Attachment model. In order to remain close to our directed network settings, we had to adapt the original model. Drawing inspiration from[12], we construct a growing random network model that exhibit scale-free properties for both indegree and outdegree measures (see Supplementary Fig. 6).

Concretely, we start from two nodes mutually connected (with weight 1). At every time step $n \geq 3$, we introduce a newborn node n and we select $m_{in} = 2$ different agents among the existing $n-1$ agents. The selection process is ruled by the preferential attachment mechanism applied to the *outdegree* of the nodes. In other words, agents with higher outdegree are more likely to be selected. Then, the newborn receives m_{in} incoming connections (of weight 1) from the selected agents. Before moving to the next time step, the newborn selects $m_{out} = 2$ different agents (among the other $n-1$) to send her outgoing ties (of weight 1). Again, the selection process follows the preferential attachment rule, though it is now based on the *indegree*.

In our test, we simulate 50 realizations of the Preferential Attachment model just described. For each of them, we run 200 time steps and we observe the behavior of the newborn agents at 8 different time-steps, namely $\{3,4,5,10,20,50,100,200\}$. Since the computational complexity of the behavior estimation method increases with the number of agents, in order to study the behavior of each observed agent n we use a reduced discrete action space

$$\tilde{\mathscr{A}} = \left\{\mathbf{a}_n \in \{0,1\}^{n-1}, \text{ s.t. } |\mathbf{a}_n|_1 \leq m_{out}\right\}.$$

In other words, we assume the newborn agent n can choose among all the combinations of (at maximum) m_{out} outgoing edges (of

	$\hat{\theta}_1$	$\hat{\theta}_1 \pm$ 95% CI	$\hat{\theta}_2$	$\hat{\theta}_2 \pm$ 95% CI	$\hat{\theta}_3$	$\hat{\theta}_3 \pm$ 95% CI
Branch-M.	1.2138		0		−1.002	
Serv.-Ad.-3	2.0205		0		0.4139	
Teller-1	0		0		0.159	
Teller-2	0.1264		0.2527		0.3212	
Teller-3	0.8664		0.8664		0.3639	
Teller-4	2.3857		2.3857		0.3555	
Teller-5	0		0		0.3752	
Teller-6	0		0		0.3136	

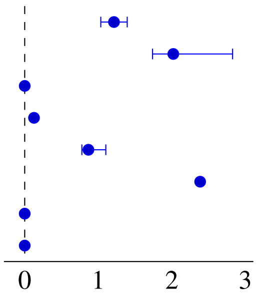

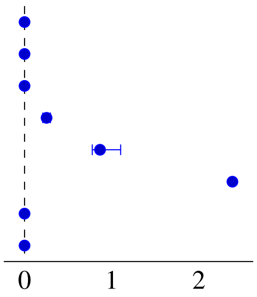

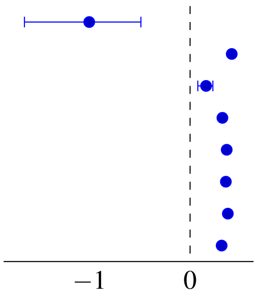

Supplementary Table 2. Australian bank estimates resulting from the Discrete Minimum-NE distance problem. Confidence intervals (CI) are built according to the behavior estimation method using a regular mesh of $n = 2^{N-1}$ samples in the action space $\mathscr{A}$, where $N = 11$ agents. Note that some of the estimates of $\hat{\theta}_1$ and $\hat{\theta}_2$ are constrained to their minimum value 0 (e.g., see Teller 1). The non-negativity constraints derive from our modelling assumptions and, unfortunately, prevent building confidence intervals for the parameters that eventually hit them (see the discussion on [39]). Yet, from a statistical inference point of view, negative estimates indicate a negative correlation with the effect associated. For instance, the $\hat{\theta}_2$ estimates suggest that almost all the agents (except for Tellers 3 and 4) strongly withstand cyclic structures, in accordance with[11] that showed how cycles are atypical structures in hierarchical networks. Note, though, that in the cases of Tellers 3 and 4 the estimates of $\hat{\theta}_2$ rely only on the "indegree" times "reciprocity" contribution, thus they cannot be distinguished from the estimate of the reciprocity itself $\hat{\theta}_1$.
Source data are provided as a Source Data file.

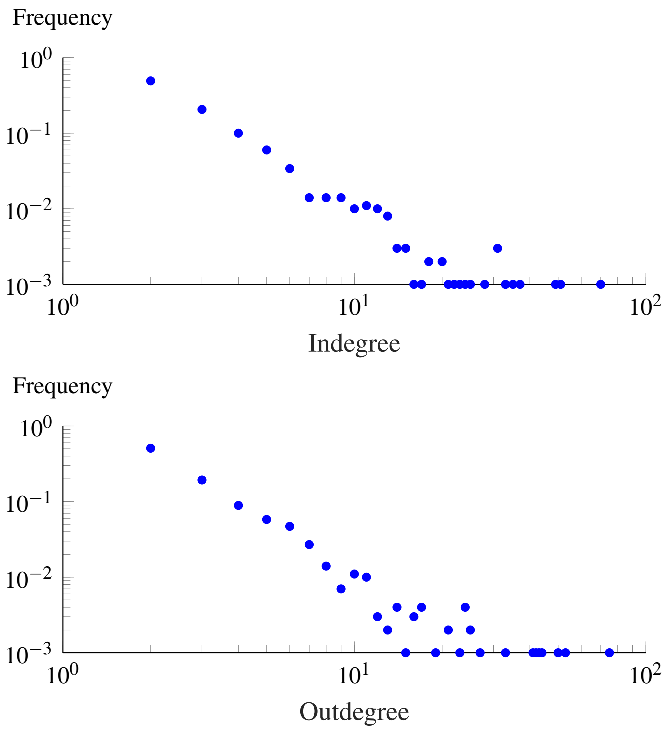

Supplementary Figure 6. Indegree and Outdegree distributions of a network of 1000 agents built with the directed Preferential Attachment model.

weight 1). We emphasize that, with this choice, we guarantee the behavior estimation method can capture all the sociological phenomena of interests, e.g., reciprocity, clustering, cyclic structures. With this extra assumption, the discrete NE-distance reads as:

$$\tilde{d}_n(\theta_n) = \left(\int_{\tilde{\mathscr{A}}} e_i^+(\mathbf{a}_i, \theta_i)^2 d\mathbf{a}_i \right)^{1/2}.$$

Finally, we would like to comment on the discretization of the parameter space, which applies to the analysis of both Preferential Attachment and Small-World models. In both cases, we restrict the parameter space to $\tilde{\Theta} = [0,2] \times 0 \times [-2,2]$, mainly for illustration purposes. In the Small-World test, this allows to capture the transitions of the NE-distance function in the parametric landscape for different model specifications (the rewiring probability p and the average indegree k). In the Preferential Attachment test, the reduced parameter space allows to conveniently color map the estimates.

To conclude, we declare that the code that performs the behavior estimation method as well as the data-sets discussed and the tests of the random network models are available at the following public repository:
https://git.ee.ethz.ch/pagann/learning_strategic_behavior.

References

1. Harker, P. T. & Pang, J.-S. Finite-dimensional variational inequality and nonlinear complementarity problems: a survey of theory, algorithms and applications. *Math. programming* **48**, 161–220 (1990).

2. Mas-Colell, A., Whinston, M. D., Green, J. R. *et al. Microeconomic theory*, vol. 1 (Oxford university press New York, 1995).

3. Esfahani, P. M., Shafieezadeh-Abadeh, S., Hanasusanto, G. A. & Kuhn, D. Data-driven inverse optimization with imperfect information. *Math. Program.* **167**, 191–234 (2018).

4. Heuberger, C. Inverse combinatorial optimization: A survey on problems, methods, and results. *J. combinatorial optimization* **8**, 329–361 (2004).

5. Ahuja, R. K. & Orlin, J. B. Inverse optimization. *Oper. Res.* **49**, 771–783 (2001).

6. Iyengar, G. & Kang, W. Inverse conic programming with applications. *Oper. Res. Lett.* **33**, 319–330 (2005).

7. Bartle, R. G. & Bartle, R. G. *The elements of integration and Lebesgue measure*, vol. 27 (Wiley Online Library, 1995).

8. Schrijver, A. *Combinatorial optimization: polyhedra and efficiency*, vol. 24 (Springer Science & Business Media, 2003).

9. Myers, R. H. & Myers, R. H. *Classical and modern regression with applications*, vol. 2 (Duxbury press Belmont, CA, 1990).

10. Pattison, P., Wasserman, S., Robins, G. & Kanfer, A. M. Statistical evaluation of algebraic constraints for social networks. *J. mathematical psychology* **44**, 536–568 (2000).

11. Davis, J. A. Clustering and hierarchy in interpersonal relations: Testing two graph theoretical models on 742 sociomatrices. *Am. Sociol. Rev.* 843–851 (1970).

12. Bollobás, B., Borgs, C., Chayes, J. & Riordan, O. Directed scale-free graphs. In *Proceedings of the fourteenth annual ACM-SIAM symposium on Discrete algorithms*, 132–139 (Society for Industrial and Applied Mathematics, 2003).
