# Supplementary material for: Game theoretical inference of human behavior in social networks
Source: Nat Commun. 2019 Dec 3;10:5507. doi: 10.1038/s41467-019-13148-8 (PMC6890725; doi:10.1038/s41467-019-13148-8)
Supplement: Supplementary file 3 — Reporting Summary [file 41467_2019_13148_MOESM3_ESM.pdf]

nature research

Corresponding author(s): DBPR NCOMMS-18-35747B

Last updated by author(s): Aug 27, 2019

Reporting Summary

Nature Research wishes to improve the reproducibility of the work that we publish. This form provides structure for consistency and transparency in reporting. For further information on Nature Research policies, see Authors & Referees and the Editorial Policy Checklist .

Statistics

For all statistical analyses, confirm that the following items are present in the figure legend, table legend, main text, or Methods section.

n/a	Confirmed	
☐	☒	The exact sample size (*n*) for each experimental group/condition, given as a discrete number and unit of measurement
☒	☐	A statement on whether measurements were taken from distinct samples or whether the same sample was measured repeatedly
☐	☒	The statistical test(s) used AND whether they are one- or two-sided<br>*Only common tests should be described solely by name; describe more complex techniques in the Methods section.*
☒	☐	A description of all covariates tested
☒	☐	A description of any assumptions or corrections, such as tests of normality and adjustment for multiple comparisons
☒	☐	A full description of the statistical parameters including central tendency (e.g. means) or other basic estimates (e.g. regression coefficient) AND variation (e.g. standard deviation) or associated estimates of uncertainty (e.g. confidence intervals)
☒	☐	For null hypothesis testing, the test statistic (e.g. *F*, *t*, *r*) with confidence intervals, effect sizes, degrees of freedom and *P* value noted<br>*Give P values as exact values whenever suitable.*
☒	☐	For Bayesian analysis, information on the choice of priors and Markov chain Monte Carlo settings
☒	☐	For hierarchical and complex designs, identification of the appropriate level for tests and full reporting of outcomes
☒	☐	Estimates of effect sizes (e.g. Cohen's *d*, Pearson's *r*), indicating how they were calculated

Our web collection on statistics for biologists contains articles on many of the points above.

Software and code

Policy information about availability of computer code

Data collection	We did not collect any data.
Data analysis	To analyze the data we used Matlab R2018a. We implemented the behavior estimation method as described in the paper. The code is available at the following link: https://git.ee.ethz.ch/pagann/learning_strategic_behavior as well as at the following address https://doi.org/10.3929/ethz-b-000360647

For manuscripts utilizing custom algorithms or software that are central to the research but not yet described in published literature, software must be made available to editors/reviewers. We strongly encourage code deposition in a community repository (e.g. GitHub). See the Nature Research guidelines for submitting code & software for further information.

Data

Policy information about availability of data

All manuscripts must include a data availability statement. This statement should provide the following information, where applicable:

- Accession codes, unique identifiers, or web links for publicly available datasets
- A list of figures that have associated raw data
- A description of any restrictions on data availability

The data used (not collected by us) on the Australian bank data set are available here: http://moreno.ss.uci.edu/data.html#bank, http://moreno.ss.uci.edu/bank.dat
The data used on the Medici network example are also not collected by us. The references are explicitly cited in the paper, and publicly available.

Field-specific reporting

Please select the one below that is the best fit for your research. If you are not sure, read the appropriate sections before making your selection.

Behavioural & social sciences study design

All studies must disclose on these points even when the disclosure is negative.

Study description	We study the strategic behavior of the individuals deducing it from their linking decisions. Our model is applied to two data-sets. The first data-set concerns the the network of marriage and business connections among Florentine families in the fifteenth century, originally collected by Kent (Kent, D. V. The rise of the Medici: Faction in Florence, 1426-1434 (Oxford University Press, USA, 1978)), but coded by Padgett and Ansell (Padgett, J. F. & Ansell, C. K. Robust action and the rise of the medici, 1400-1434. Am. journal sociology 98, 1259–1319 (1993)). The second data-set concerns the confiding relationships within an Australian bank. The data-set is taken from: "Pattison, P., Wasserman, S., Robins, G. & Kanfer, A. M. Statistical evaluation of algebraic constraints for social networks, J. mathematical psychology (2000)". We store the data we analyzed at the following address ''https://doi.org/10.3929/ethz-b-000360648''.
Research sample	The research sample is taken from employees of an Australian bank and from Renaissance Florentine families. We did not collect data, which simply used the data published.
Sampling strategy	We did not perform the sampling strategy.
Data collection	We did not collect data.
Timing	We did not collect data.
Data exclusions	No data were excluded from the analysis.
Non-participation	We did not collect data.
Randomization	We did not collect data.

Reporting for specific materials, systems and methods

We require information from authors about some types of materials, experimental systems and methods used in many studies. Here, indicate whether each material, system or method listed is relevant to your study. If you are not sure if a list item applies to your research, read the appropriate section before selecting a response.

Materials & experimental systems

n/a	Involved in the study	
☒	☐	Antibodies
☒	☐	Eukaryotic cell lines
☒	☐	Palaeontology
☒	☐	Animals and other organisms
☐	☒	Human research participants
☒	☐	Clinical data

Methods

n/a	Involved in the study	
☒	☐	ChIP-seq
☒	☐	Flow cytometry
☒	☐	MRI-based neuroimaging

Human research participants

Policy information about studies involving human research participants

Population characteristics	We did not collect data
Recruitment	We did not collect data
Ethics oversight	We did not collect data

Note that full information on the approval of the study protocol must also be provided in the manuscript.
